# Bamboo‐Slip‐Inspired Conformal Reconfigurable Metasurfaces

**DOI:** 10.1002/advs.77188

**Published:** 2026-08-13

**Authors:** Qifan Li, Shaofeng Zhang, Qiang Feng, Dexiao Xia, Yajie Mu, Xiangjin Ma, Jiaqi Han, Haixia Liu, Guangyao Liu, Long Li

**Affiliations:** ^1^ School of Electronic Engineering Key Laboratory of High Speed Circuit Design and EMC of Ministry of Education Xidian University Xi'an China; ^2^ State Key Laboratory of Terahertz and Millimeter Waves and Department of Electrical Engineering City University of Hong Kong Hong Kong China

**Keywords:** Bamboo‐slip‐inspired splicing subarray, conformal metasurfaces, reconfigurable pattern, wide angle scanning

## Abstract

Metasurfaces with reconfigurable shapes have a stronger adaptability to complex environments. This paper designs a bamboo‐slip‐inspired conformal reconfigurable transmission metasurface system with splicing ability and scalability. Bamboo‐slip‐inspired subarrays are assembled on different support skeletons to form conformal metasurfaces of various shapes to adapt to wide‐angle beam coverage in different environments. In order to realize the switching of radiation domain, a pattern reconfigurable antenna as feed source is designed in this paper. The radiation direction of the pattern reconfigurable antenna is adjusted by the turning on or off of the PIN diode. To verify the proposed conformal metasurface properties, a prototype was fabricated. The prototype demonstrates the powerful beam control capabilities of cylindrical, semi‐hexagonal, and parabolic surfaces, respectively. The bamboo‐slip‐inspired conformal reconfigurable metasurface can be deployed on the surface of communication platforms of different shapes to adapt to various complex curved structures or space constraints, and can be well integrated with devices or systems.

## Graphical abstract

Bamboo‐slip‐inspired conformal reconfigurable metasurface system. The structure is mainly composed of three parts, which are conformal metasurface, pattern reconfigurable antenna, and control components (such as Field Programmable Gate Array (FPGA)). Conformal metasurfaces draw inspiration from the design of ancient Chinese bamboo slips.

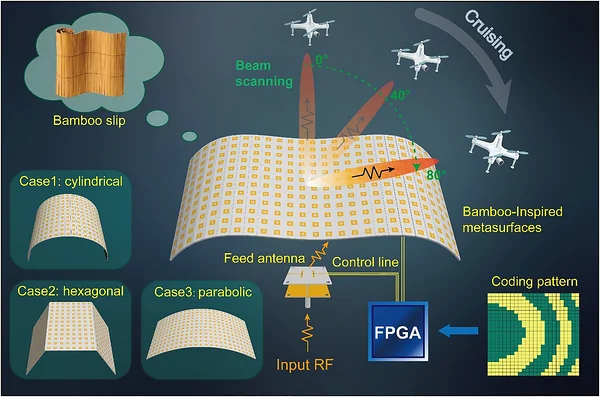

## 1 Introduction

Phased array antennas is widely used in radar detection, communication, remote sensing mapping and biomedical fields because of its flexible beam control ability [1, 2, 3, 4]. However, complex transmitter and receiver (T/R) components make the phased array antenna system complicated, huge in size, and extremely costly [5, 6]. In recent years, the proposal of reconfigurable metasurfaces has perfectly addressed the aforementioned shortcomings of phased array antenna [7, 8, 9, 10, 11]. Because metasurfaces adopt spatial feeding, beamforming can be achieved without a complex phase shift network, greatly simplifying the complexity of the system and processing costs [12, 13, 14]. Due to simple structure and easy integration, reconfigurable metasurfaces have been widely applied in 5G communication, wireless power transfer (WPT), orbital angular momentum (OAM), holographic imaging, etc [15, 16, 17, 18, 19, 20, 21]. However, reconfigurable metasurfaces based on planar shapes often struggle to adapt to complex and ever‐changing environments.

In recent years, the research on conformal metasurfaces has gradually received attention to adapt to different working scenarios [22, 23, 24, 25, 26, 27]. Due to excellent aerodynamic performance, conformal metasurfaces have been widely applied to curved platforms such as aircraft and missiles. Compared with planar metasurfaces, conformal metasurfaces have structural advantages such as structurally integrated, provides extra space, and having no drag [28, 29, 30]. In addition, conformal metasurfaces also exhibit many advantages in electrical performance. First, the gain of a planar metasurface drops with increased scan. However, the gain of a conformal metasurface is controlled and depends on its shape [31]. Second, the angular coverage of planar metasurfaces is limited roughly to ± 60°, but the conformal metasurfaces is very wide, even half sphere [32, 33]. A cylindrical array can even achieve 360° omnidirectional angular coverage [34]. Finally, compared with planar metasurfaces, conformal metasurfaces have a wider frequency bandwidth and a smaller radar cross‐section (RCS) [35, 36].

However, at present, most conformal metasurfaces are rigid conformal structures [31, 35, 37, 38, 39, 40]. Once the design and fabrication are completed, the shape of its structure cannot be changed. To obtain the shape of the conformal metasurface we desire, it is usually necessary to apply external force to the planar metasurface to bend it [41]. This requires the electronic components on the metasurface to have high soldering strength. In addition, the mechanical bending method of conformal metasurfaces will result in the metasurfaces not being too thick [42]. This severely restricts the design freedom of conformal metasurfaces. Considering the conflict between rigid structures and conformal forms, some researchers proposed using flexible structures to achieve the conformal effect of metasurfaces [28, 43, 44, 45, 46, 47, 48, 49, 50]. The dielectric substrate is made of flexible materials, and the metasurface can bend within a certain curvature. However, flexible materials offer only a limited range of dielectric constants, which leads to the impairment of the design freedom of conformal metasurfaces [51].

To adapt to the shape requirements of different environments and make full use of the existing printed circuit board (PCB) technology, this paper adopts the design idea of the splitable subarrays. The splitable reconfigurable conformal metasurface is composed of rigid planar splitable subarrays and flexible structural skeletons. Such a design has the advantages of easy processing, low cost, high bendable curvature, and the reusability of subarrays. Meanwhile, the thickness of the dielectric plate of the subarray is no longer affected by 3D printing technology or conformal curvature [52, 53] It is worth noting that the inspiration for the design comes from ancient Chinese bamboo slips. Therefore, this design is also named bamboo‐slip‐inspired conformal reconfigurable metasurfaces.

## 2 Results

The designed bamboo‐slip‐inspired metasurface system is shown in Figure 1. The structure is mainly composed of three parts, which are conformal metasurface, pattern reconfigurable antenna, and control components (such as Field Programmable Gate Array (FPGA)). Conformal metasurfaces draw inspiration from the design of ancient Chinese bamboo slips. It is designed in a long strip shape with independent control. Multiple identical bamboo slips can be combined into different shapes, such as semi‐cylindrical, semi‐hexagonal and parabolic cylindrical shapes. Each metasurface of the bamboo slips is composed of 12 independently controlled 1‐bit transmissive reconfigurable meta‐atom. Just as different characters on bamboo slips convey different information, different PIN diode states on the bamboo‐slip‐inspired metasurface can be used to control different electromagnetic wavefronts, such as the beam pointing angle shown in Figure 1.

**FIGURE 1:**
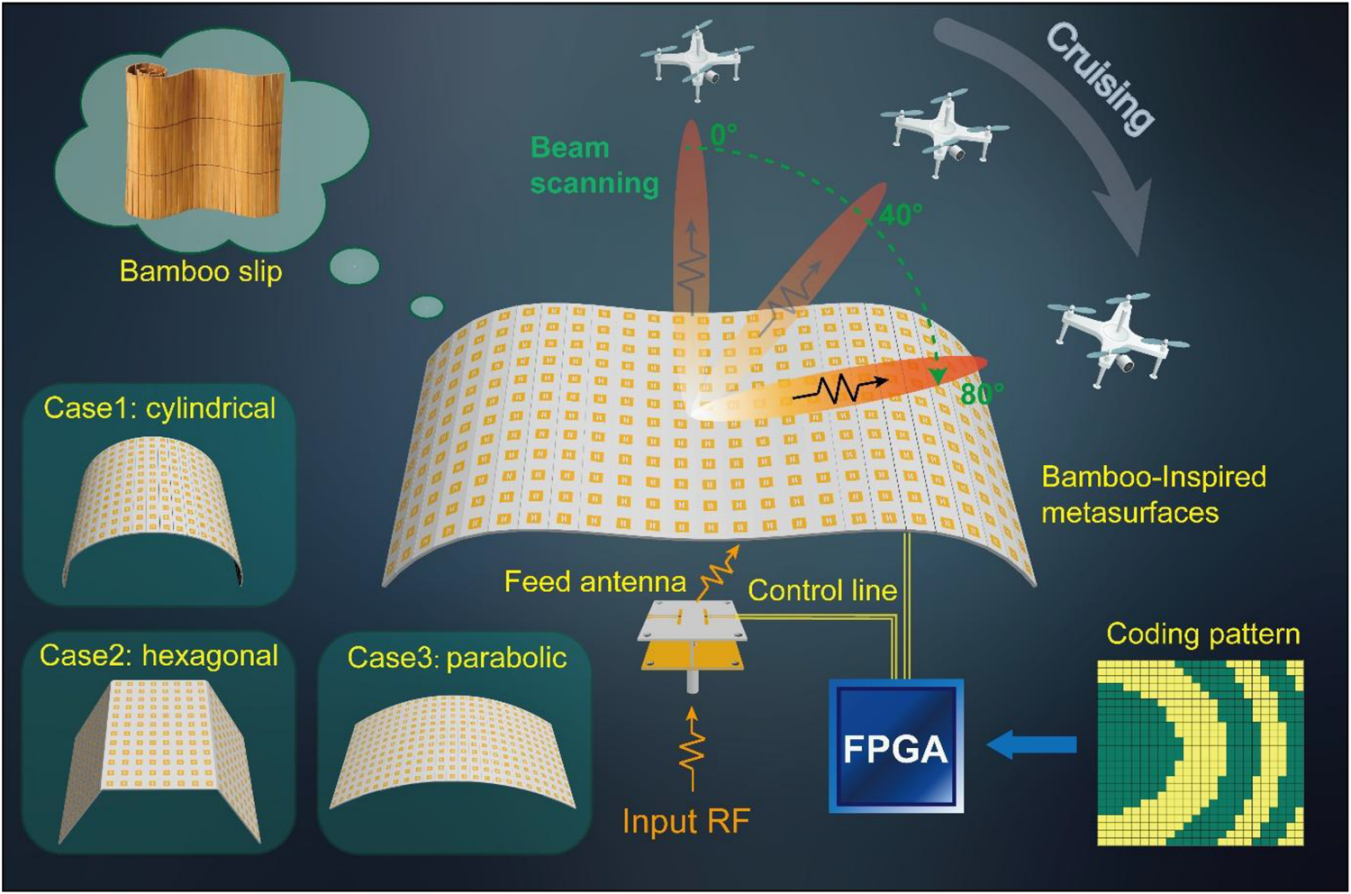
Bamboo‐slip‐inspired conformal reconfigurable metasurface system.

The pattern reconfigurable antenna is used as a feed antenna in this system. When the metasurfaces of bamboo slips are spliced into a curved surface, the feed source needs to constantly switch the radiation area so that the array surface utilization rate of the bamboo slip metasurface can be optimized, as shown in Note S1 and Figure S1. The pattern reconfigurable antenna consists of a vertical dipole antenna and a pair of reconfigurable transducers. The different PIN diode states on the leader cause the antenna to have different pattern directions, that is, to achieve the purpose of changing the radiation area. FPGA and control components are mainly used to change the state of the PIN diode of the bamboo‐slip‐inspired metasurface and the pattern reconfigurable antenna, thereby efficiently transmitting electromagnetic information.

### 2.1 Meta‐Atom Design

The proposed meta‐atom adopted are classic U‐shaped and O‐shaped slot structures [54], as shown in Figure 2a. Design principles and details of the meta‐atom are given in Note S2. The meta‐atom consists of three metal layers, two dielectric substrates and a bonding film layer. The metal layers, from top to bottom, are the radiation structure layer (upper patch), metal ground (GND) and receiving structure layer (down patch). The dielectric substrates used are Shengyi S7136H with a dielectric constant of 3.55. The thickness of both dielectric substrates is 1.524 mm. The bonding film layer used is Rogers 4450F and its thickness is 0.4 mm. The radiation patch consists of an O‐shaped slot structures, and the inner side is connected by a strip loaded with PIN diodes. The PIN diode is SMP1340. By adjusting the states of the PIN diodes, 1‐bit phase modulation for transmission can be achieved, as shown in Figure S2e. Detailed design parameters of the meta‐atom are provided in Table S1 (Supporting Information).

**FIGURE 2:**
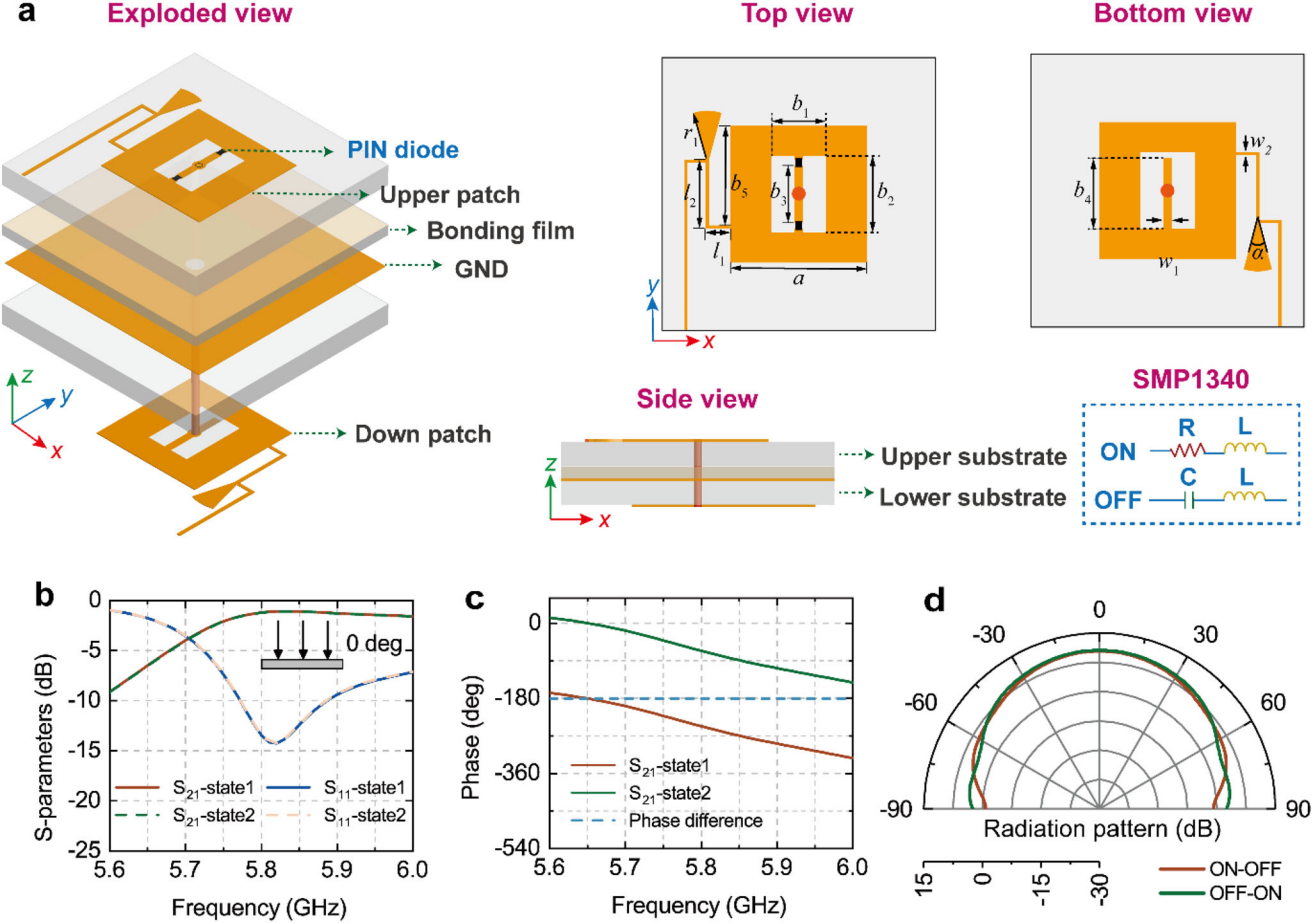
Design of the meta‐atom. (a) 3D and 2D structures. (b) amplitude and (c) phase characteristics. (d) Radiation pattern in a periodic array.

The proposed meta‐atom under periodic boundary conditions was simulated in the commercial software Ansys High Frequency Structure Simulator (HFSS) 2021. The radiation and transmission characteristics of the meta‐atom are shown in Figure 2. The transmission coefficients and phases of these two states (1‐bit) at normal incidence are shown in Figure 2b,c. The operating bandwidth is defined as the frequency range over which the phase difference between the two states remains within 180° ± 10° while the transmission loss of both states is better than –1 dB (i.e., |S_21_| > –1 dB), yielding a bandwidth of 5.77–5.89 GHz. Within this band, the phase difference is maintained at 180° with a deviation within ±2°. Although this amplitude‐defined bandwidth is somewhat narrower than that of some reported designs, bandwidth is not the primary focus of this work [55]. Instead, our design prioritizes wide‐angle scanning stability and low insertion loss under oblique incidence, which are essential for conformal beam‐scanning applications. As demonstrated in Figure S3 in Note S2, the meta‐atom maintains an insertion loss below 1.21 dB and a phase error within 2° up to 40° oblique incidence. In addition, Figure 2d presents the H‐plane of radiation pattern in a periodic array for the on‐off state (state 1) and off‐on state (state 2). The radiation patterns of the two states are nearly identical and vary gently with angle, a feature that fully enables the beam scanning capability of the metasurface array.

### 2.2 Feed Source Design

To achieve dynamic switching of the feed source illumination area, we designed a pattern reconfigurable antenna, as shown in Figure 3. The pattern reconfigurable antenna consists of a vertical dipole antenna and two active guiding elements at the top. The vertical dipole antenna is printed on the dielectric substrates perpendicular to the metal ground (GND) and fed via the Γ‐shaped microstrip feeder. The dielectric substrate perpendicular to GND is fixed on the metal ground through mounting holes. Active guiding elements in dipole shapes are printed on the top horizontal dielectric substrate. the PIN diode is loaded on the active guiding element. The PIN diode is also SMP1340. All the dielectric substrates used are FR4 with a dielectric constant of 4.4. The thickness of both dielectric substrates is 1 mm. Feed the pattern reconfigurable antenna using the coaxial line. The metal wires on both sides of the guide are DC feedlines. The function of the inductance on the feedlines is to block the AC signal. There are four states (On‐On, On‐Off, Off‐On and Off‐Off) in total for the combination of two PIN diodes. Among them, the On‐On state is not available. Detailed design parameters of the pattern reconfigurable antenna are shown in Figure S4 and Table S2. The operating principle of the pattern‐reconfigurable antenna, based on the Yagi‐Uda antenna, is provided in Figure S5 in Note S3.

**FIGURE 3:**
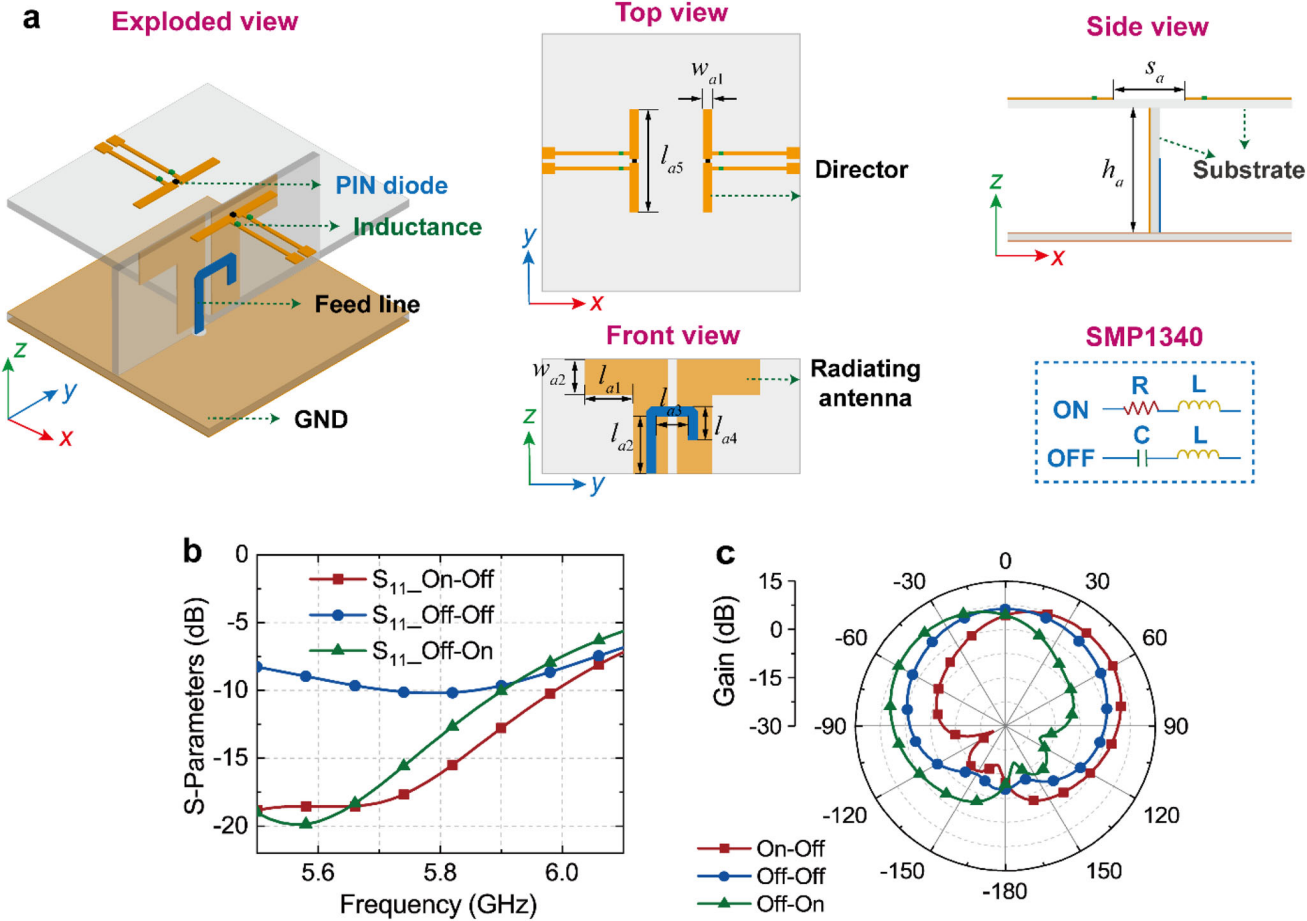
Design of the feed antenna. (a) 3D and 2D structures. (b) Reflection coefficients in different states. (c) Radiation pattern in different states.

The designed pattern reconfigurable antenna was simulated in HFSS. The reflection coefficient and radiation pattern are shown in Figure 3. The antenna operates from 5.7 to 5.9 GHz. The reflection coefficients of the three available states within the working range are all less than −10 dB, shown in Figure 3b. The 3D radiation pattern and H‐plane radiation pattern of the pattern reconfigurable antenna in different states of the PIN diode are shown in Figure S5 and Figure 3c, respectively. When state is Off‐Off, the radiation direction is the principal axis direction (*θ* = 0°), while in the other two states, the radiation direction deviates from the principal axis by 40° (*θ* = ± 40°). By switching the above three states, the purpose of switching the radiation area can be achieved.

### 2.3 System Integration: Bamboo‐Slip‐Inspired Splicing

The bamboo‐slip‐inspired conformal reconfigurable metasurface proposed in this paper has the advantages of splicing ability and flexible scalability, as shown in Figure 4. Conformal metasurfaces are composed of many sub‐arrays with the shape of bamboo‐slip. Each bamboo‐slip is composed of 12 independently controlled meta‐atoms. The specific introduction of the meta‐atoms used is shown in Figure 2. According to the predesigned curve *z* = *f* (*x*, *y*), the sub‐arrays with the shape of bamboo‐slip can be spliced into the corresponding cylinder as shown in Figure 4. Based on advantage of this design principle, we can splice various cylindrical conformal reconfigurable metasurfaces. In addition, this design can also expand the original size of the metasurface array according to the actual application requirements. Not only the metasurface, but also the corresponding FPGA board and the DC control line also adopt scalable design principles, as shown in Figure 4.

**FIGURE 4:**
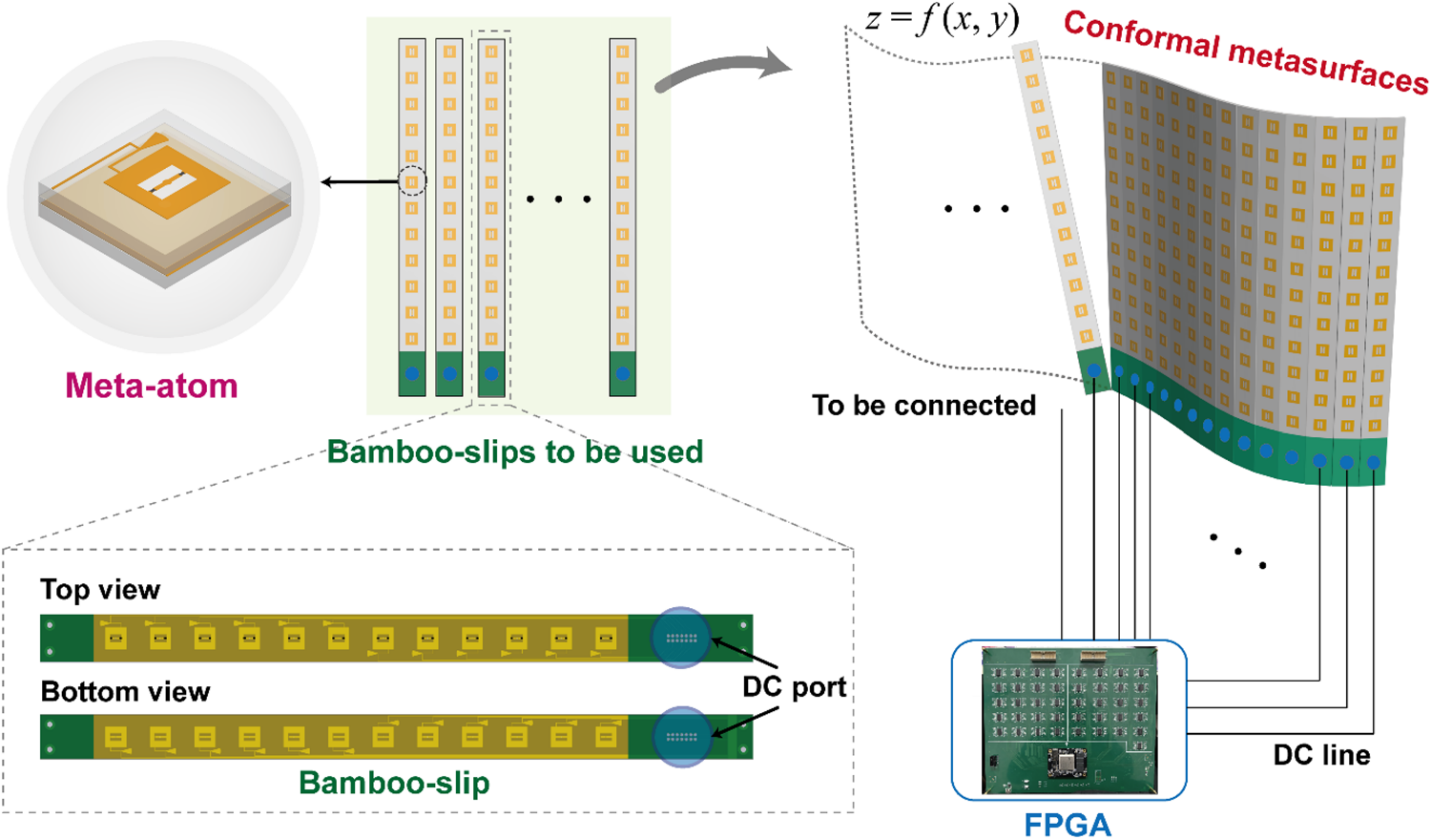
The splicing ability and scalability of bamboo‐slip‐inspired conformal reconfigurable metasurface.

### 2.4 Beamforming Theory of Conformal Metasurfaces

As shown in the Cartesian coordinate system in Figure 5b, the generatrix direction of the one‐dimensional cylindrical metasurface is along the *x*‐direction. The center coordinates of the unit cell of the conformal metasurfaces in the *m*‐th row and *n*‐th column are ${\vec{r}}_{mn}=\left(x_{mn},y_{mn},z_{mn}\right)$, and the wave vector pointing in the direction of (θ, φ) is

(1) $$\vec{k}=\frac{2\pi }{\lambda }\left(\sin\theta \cos\varphi ,\sin\theta \sin\varphi ,\cos\theta \right)$$

where λ is the wavelength in free space. The phase required for generating a pencil beam pointing at angle (θ_0_,φ_0_) is

(2) $$\begin{array}{ccc} \varphi_{0}\left(m,n\right) & = & \vec{k}\cdot {\vec{r}}_{mn}=\frac{2\pi }{\lambda }(x_{mn}\sin\theta_{0}\cos\varphi_{0}\\ & & +\;y_{mn}\sin\theta_{0}\sin\varphi_{0}+z_{mn}\cos\theta_{0}) \end{array}$$

**FIGURE 5:**
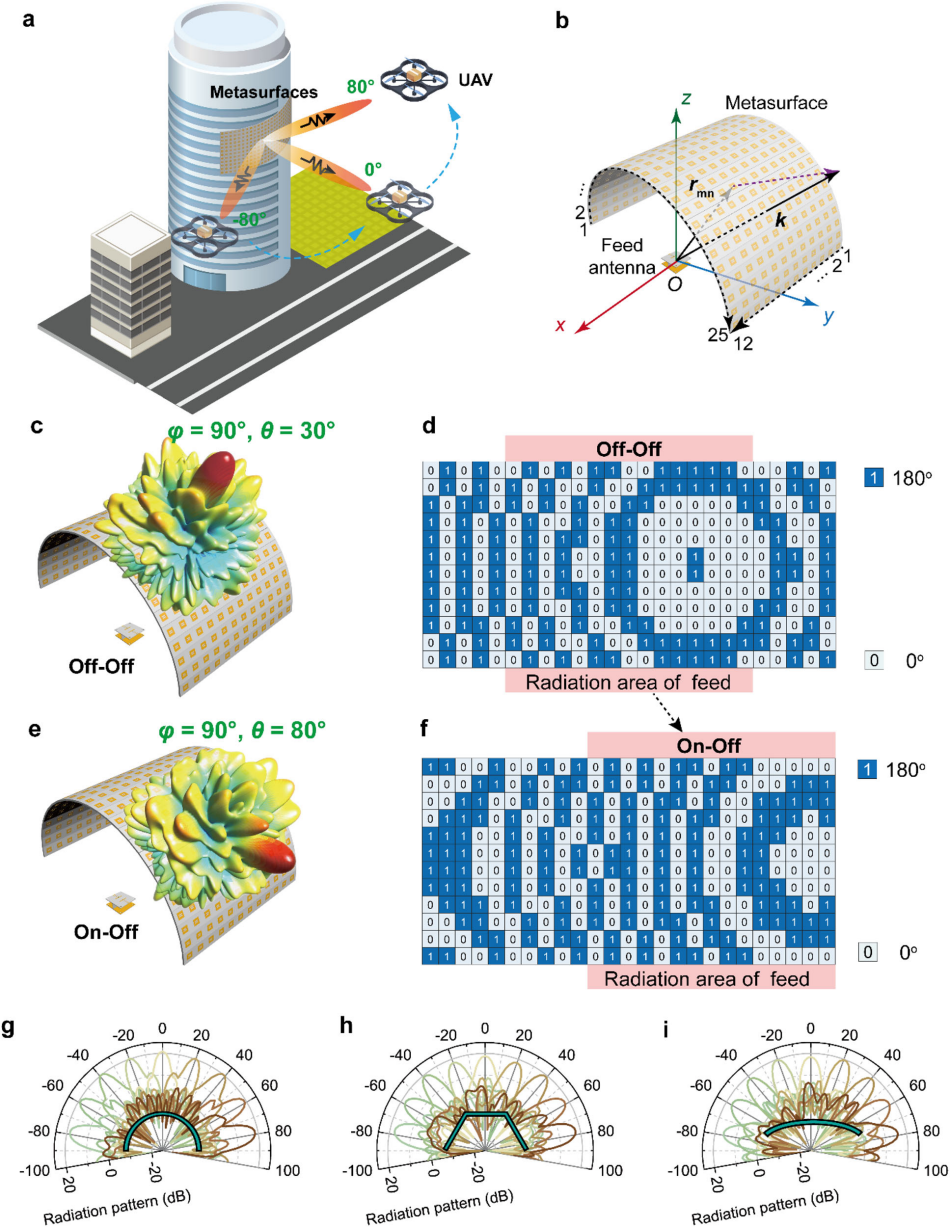
Simulation model and results scanned along the circumferential direction. (a) Application scenarios of circumferential beam scanning. (b) Composition and beamforming model of cylindrical conformal metasurfaces. (c–f) The 3D radiation pattern and encoding when the angles are [*φ* = 90°, *θ* = 30°] and [*φ* = 90°, *θ* = 80°] respectively. 2D beam scanning radiation pattern of (g) cylindrical, (h) semi‐hexagonal prisms, and (i) parabolic.

The phase center of the feed antenna is set at the origin *O*, then the spatial compensating phase between the feed antenna and the conformal metasurface is

(3) $$\varphi_{f}\left(m,n\right)=\frac{2\pi }{\lambda }\sqrt{x_{mn}^{2}+y_{mn}^{2}+z_{mn}^{2}}$$

The compensation phase of the unit cell of metasurface is

(4) $$\varphi \left(m,n\right)=\varphi_{f}\left(m,n\right)-\varphi_{0}\left(m,n\right)$$

Since the designed metasurface has a 1‐bit phase resolution, further quantization of the combined phase expression can be obtained as follows:

(5) $$\varphi_{D}\left(m,n\right)=\left\{\begin{array}{c} 180^{o},\varphi \left(m,n\right)\in \left[180^{o},360^{o}\right)\\ 0^{o},\varphi \left(m,n\right)\in \left[0^{o},180^{o}\right) \end{array}\right.$$

Of course, there is more than one calculation method for phase compensation of conformal metasurfaces. This paper provides another method in Note S4, which is based on the generalized Snell theorem of surfaces, as shown in Figures S6 and S7. For the cylindrical, semi‑hexagonal, and parabolic surfaces studied in this work, both methods yield identical phase compensation results, as they solve the same physical problem. According to Equations (1)—(5), the phase compensation of any surface can be calculated. It can be seen from Equations (2) and (3) that different surfaces only have different coordinates at the centers of the array meta‐atoms. The coordinates of the array meta‐atoms are determined by their surface equations. For example, the equation of the conformal semi‐cylindrical metasurface shown in Figure 5b is

(6) $$\left\{\begin{array}{c} z^{2}+y^{2}=R^{2},\\ x=\mathrm{constant},\\ z>0,-L/2<x<L/2, \end{array}\right.$$

where *R* is the radius of the cylindrical surface, *L* is the length of the generatrix of the cylindrical surface. For the equations concerning semi‐hexagonal prisms and parabolic prisms, refer to Figure S8 and Note S5.

### 2.5 Full‐Wave Simulation Results

The designed conformal reconfigurable metasurfaces was simulated in Ansys HFSS. The simulation environment is shown in Figure 5c,e. The boundary conditions for both the metasurface and the antenna are Finite Element‐Boundary Integral (FE‐BI). The feed antenna operating at 5.8 GHz. The system can complete beam scanning along the circumferential direction, as shown in Figure 5a. A pencil beam with a pointing angle of [*φ* = 90°, *θ* = 30°] is generated in Figure 5c. The corresponding conformal metasurface coding is shown in Figure 5d. The pattern reconfigurable antenna as the feed source is in the Off state, and its main radiation direction is 0°. The radiation area illuminated by the feed antenna onto the metasurface is shown in the pink area. When the main beam switches to a larger angle (*θ* > 50°), the feed antenna also needs to switch the radiation area. For example, a pencil beam with a pointing angle of [*φ* = 90°, *θ* = 80°] is generated, as shown in Figure 5e. The encoding of the conformal metasurface is shown in Figure 5f. To ensure that the conformal metasurface still maintains efficient radiation at large angle, the reconfigurable feed antenna will change from the Off‐Off state to the On‐Off state. This will cause the radiation area on the conformal metasurface (the pink area in Figure 5f) to shift.

Through conformal metasurface coding and the coordinated variation of the reconfigurable feed antenna radiation area, the semi‐cylindrical conformal metasurface has an ultra‐wide beam scanning capability ranging from −80° to 80°, as shown in Figure 5g. For semi‐hexagonal prisms and paraboloids, the corresponding beam scanning results are shown in Figure 5h,i. The simulation results show that the beam scanning range of the cylindrical surface is ± 80°, while the scanning ranges of the semi‐hexagonal and the parabolic prism are ± 79° and ± 80°, respectively. The main lobe gain, first side lobe, back lobe, and 3 dB half‐power beamwidth of the conformal metasurfaces of three typical shapes are shown in Figure S9 and discussed in Note S6.

In addition, since each meta‐atom of the bamboo‐slip‐inspired conformal reconfigurable metasurface is independently controlled, the system can also complete beam scanning along the generatrix direction, as shown in Figure 6a. A pencil beam with a pointing angle of [*φ* = 0°, *θ* = 0°] is generated, as shown in Figure 6b. The corresponding conformal metasurface coding is shown in Figure 6d. The reconfigurable antenna at this moment is in the Off state. If the pointing angle of the pencil beam is switched to [*φ* = 0°, *θ* = 30°], shown in Figure 6b. The changed encoding of the conformal metasurface is shown in Figure 6e, and the corresponding reconfigurable feed antenna will remain in the same state. The corresponding beam scanning results are shown in Figure 6f. For semi‐hexagonal prisms and paraboloids, the corresponding beam scanning results are shown in Figure 6g,h. The simulation results show that the beam scanning range (along the generatrix direction) of the cylindrical surface is ± 45°, while the scanning ranges of the semi‐hexagonal and the parabolic prism are ± 40° and ± 45°, respectively.

**FIGURE 6:**
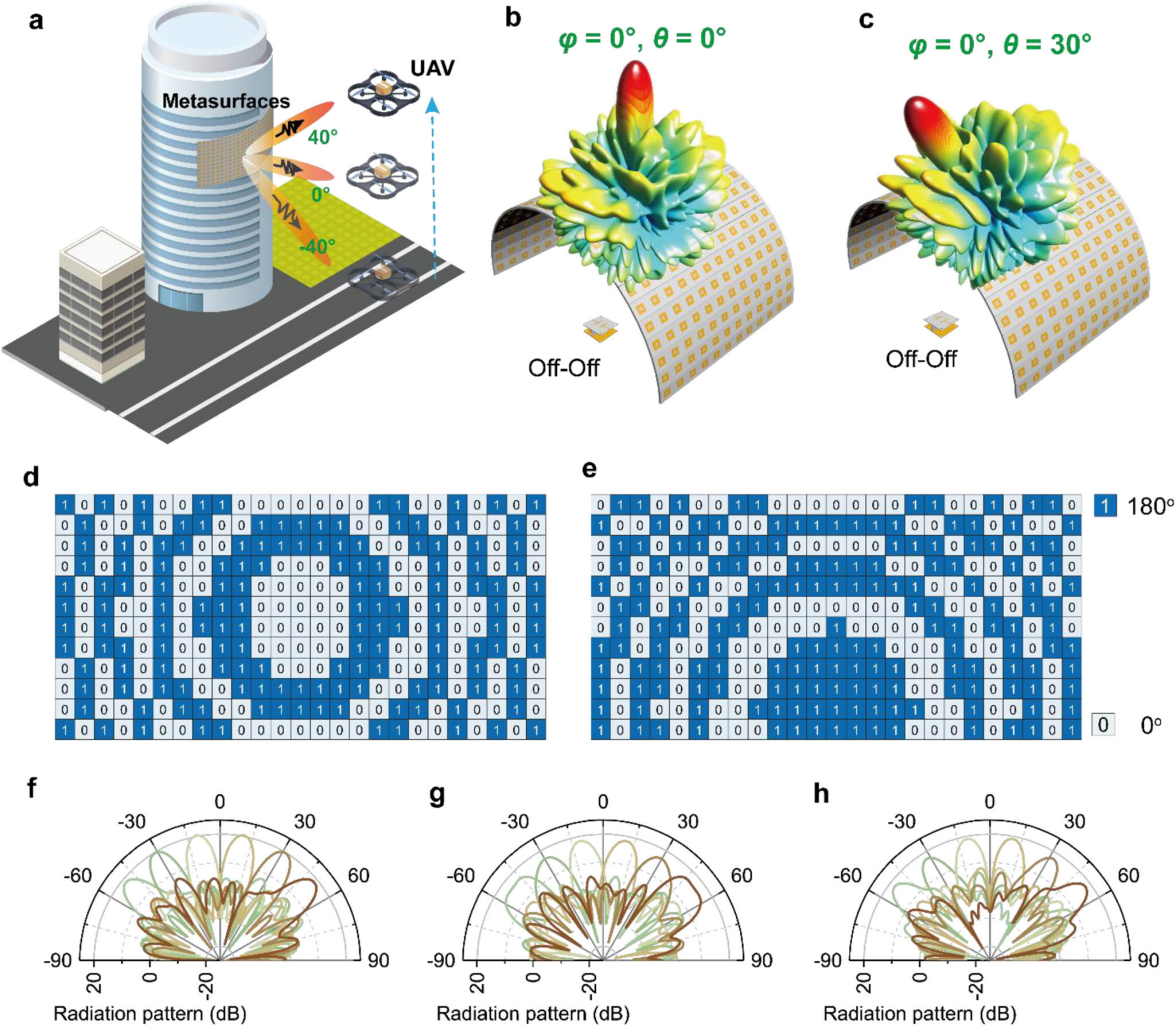
Simulation model and results scanned along the generatrix direction. (a) Application scenarios of generatrix beam scanning. (b–e) The 3D radiation pattern and encoding when the angles are [*φ* = 0°, *θ* = 0°] and [*φ* = 0°, *θ* = 30°] respectively. 2D beam scanning radiation pattern of conformal metasurfaces of (f) cylindrical, (g) semi‐hexagonal prisms, and (h) parabolic.

### 2.6 Experimental Results

The number of 25 bamboo‐slip‐inspired metasurface subarrays were fabricated, as shown in Figure 7a. They are successively connected together using 3D‐printed support skeletons to form a semi‐cylinder, a semi‐hexagonal prism and a parabolic cylinder. A detailed prototype is provided in Figure S10 in Note S7. Each subarray contains 12 meta‐atoms, and two PIN diodes need to be soldered on the top layer of each meta‐atom, as shown in Figure 7b. In addition, a pattern reconfigurable antenna was processed, as shown in Figure 7c. Two PIN diodes are soldered on each director, respectively. The back also required an SMA RF connector. The state switching of the pattern reconfigurable antenna and metasurface is both accomplished by FPGA. A detailed prototype of DC control board is provided in Figure S11 in Note S8. By controlling the on‐off state of the PIN diode on the meta‐atom through FPGA, the beam scanning of a small area can be carried out. The radiation zone switching of the feed source can be achieved by controlling the PIN diode on the reconfigurable antenna through the FPGA. These two controls can operate independently. The next step involved testing the radiation performance of the fabricated metasurface.

**FIGURE 7:**
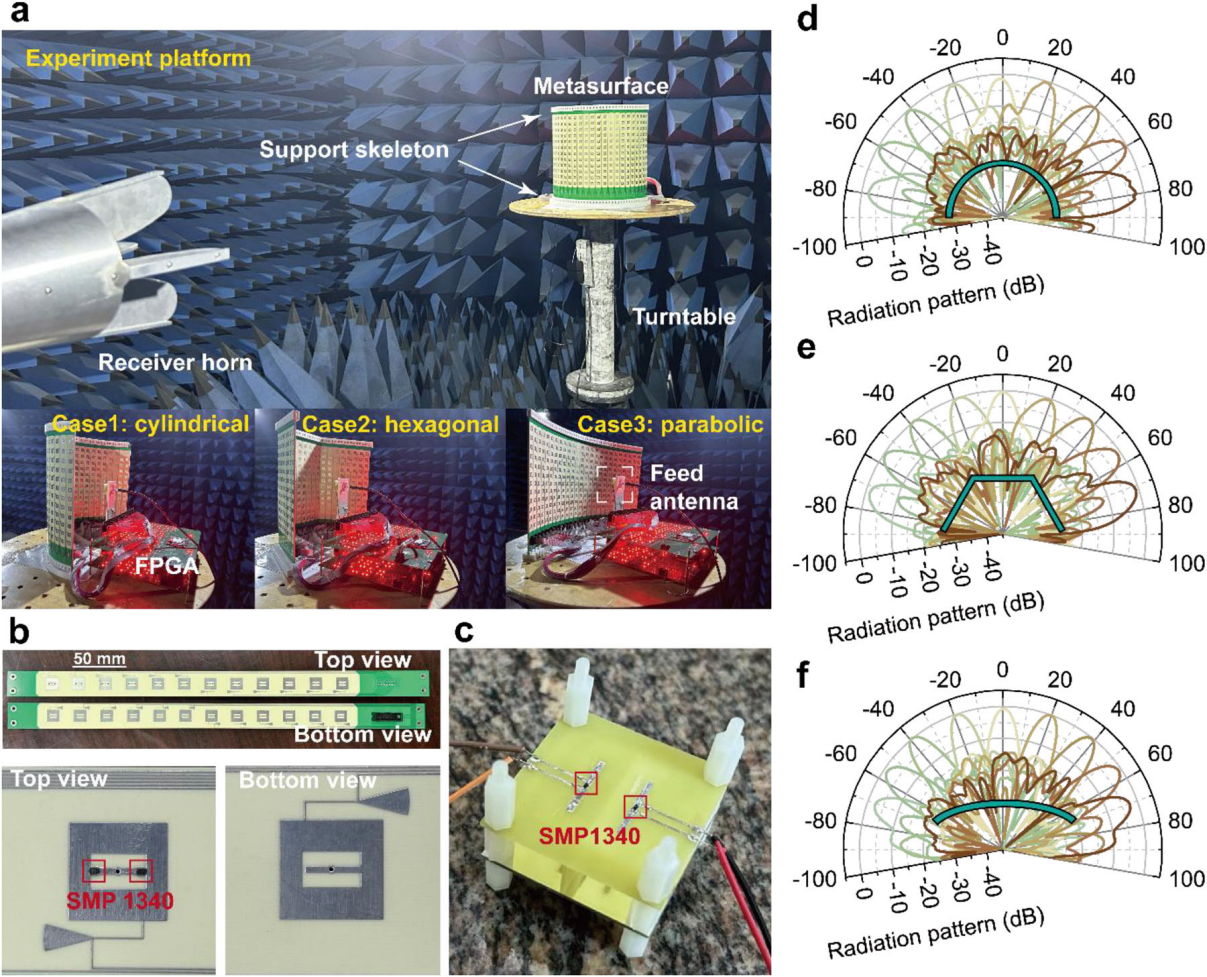
Experimental platform, prototype and results (a) Testing in the microwave anechoic chamber. Fabrication prototype of (b) metasurface and of (c) pattern reconfigurable antenna. 2D beam scanning radiation pattern of conformal metasurfaces of (d) cylindrical, (e) semi‐hexagonal prisms, and (f) parabolic.

The entire system was placed in a microwave anechoic chamber for experiments, as shown in Figure 7a. The conformal metasurface system is placed on a turntable. By rotating the turntable, its scanning radiation pattern can be tested. The experimental process of the 1‐bit meta‐atom is provided in Note S9, and the measured S‐parameter magnitude and phase are depicted in Figure S12c,d. The S‐parameters and two‐dimensional (2D) radiation pattern of the pattern reconfigurable antenna are shown in Figure S13 in Note S10. The two‐dimensional radiation direction semi‐cylinder, semi‐hexagonal prisms, and parabolic prism are shown in Figure 7d–f. The experimental results show that the beam scanning range of the cylindrical surface is ± 80°, while the scanning ranges of the semi‐hexagonal and the parabolic prism are ± 76° and ± 75°, respectively. The gain, first side lobe, rear lobe, and 3 dB half‐width of the conformal metasurfaces of three typical shapes can be found in Figure S14 in Note S11.

It should be noted that, for both simulated and experimental results, the gain fluctuation over the ±80° scanning range remains within 3 dB for the cylindrical and semi‐hexagonal prismatic surfaces. In contrast, the parabolic surface exhibits a gain fluctuation exceeding 3 dB when the scan angle deviates beyond ±60°. This behavior is mainly due to the aperture utilization efficiency inherent to different curved geometries, as detailed in Figure S15 and Note S12. The parabolic profile employed in this work features a relatively small curvature, rendering its effective aperture utilization efficiency versus *θ* very similar to that of a planar aperture, which typically experiences a gain drop of more than 3 dB at ±60° scanning, as shown in Figure S16. To mitigate this gain fluctuation for the parabolic configuration, two viable approaches are the optimization of the feed excitation scheme or the modification of the parabolic curvature.

The overall beam switching time of the metasurface system is less than 300 ns, making it applicable to millisecond‐response drone‐enabled dynamic real‐time communication scenarios, as discussed in Note S13, and the switching times of the specific hardware are listed in Table S3. The total power consumption of the system is approximately 3.5–4.4 W. The power consumption of the specific components is listed in Table S4, and the corresponding calculation and analysis are provided in Note S14.

## 3 Discussions

### 3.1 Analysis of Inter‐Facet Junctions

For quasi‐curved arrays composed of multiple planar facets, the analysis and design of inter‐facet junctions represent one of the most challenging issues in conformal array theory. For wide gaps induced by significant curvature, the edges of subarrays and the gaps between adjacent subarrays exhibit strong diffraction characteristics. Therefore, we have conducted a focused analysis on the gap configurations employed in our design, and evaluated their impact on both the main lobe and side lobe performance, as shown in Figure 8. As shown in Figure 8a, gaps exist between adjacent subarrays in the fabricated prototype. These gaps are sequentially filled with dielectric material, resulting in a piecewise‐smooth conformal surface, as shown in Figure 8b. Figure 8c and Table 1 compare the radiation patterns, side lobe levels, and gains for three curved metasurfaces with and without gaps. The results confirm that the gap effects are within acceptable tolerances. For the cylindrical profile, the gain and side lobe errors are only 0.1 dB and 0.25 dB, respectively, indicating negligible influence. For the semi‐hexagonal profile, the gain error is 0.51 dB while the side lobe error is −2.01 dB, suggesting a beneficial suppression of side lobes. For the parabolic profile, the errors are 0.25 and 0.66 dB, respectively, confirming a minor and acceptable impact.

**FIGURE 8:**
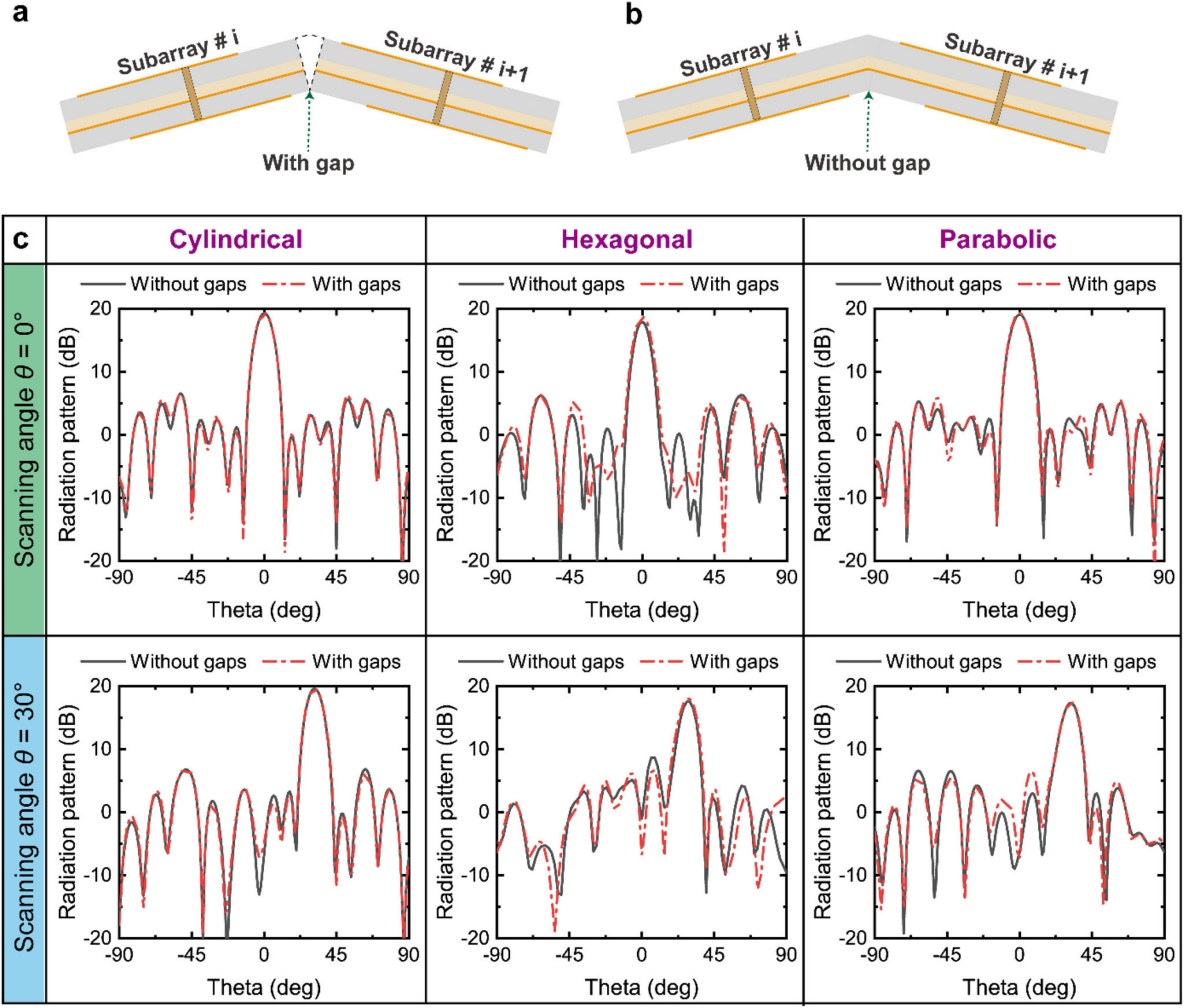
Conformal metasurface inter‐facet junctions: (a) with gaps, (b) without gaps, and (c) effect on scanning beam.

**TABLE 1:** Effect of inter‐facet junctions on gain and side lobes.

	Gain (dB)		Side lobes (dB)	
Scanning angle	*θ* = 0°	*θ* = 30°	*θ* = 0°	*θ* = 30°
Cylindrical	19.28 (19.29)	19.58 (19.68)	6.54 (6.43)	6.83 (7.08)
Hexagonal	17.88 (18.15)	17.51 (18.02)	6.31 (6.36)	8.69 (6.68)
Parabolic	19.04 (19.29)	17.35 (17.51)	5.22 (5.88)	6.47 (6.42)

*Note*: Values in parentheses are simulated results with gaps, while values outside parentheses are simulated results without gaps.

Overall, all three conformal metasurfaces exhibit stable gain and side lobe performance across the two scanning angles, with the maximum gain variation confined to within 0.6 dB and side lobe levels ranging from 5 to 9 dB across all cases. These quantitative findings verify that the coupling and diffraction effects induced by the gaps in our prototype do not appreciably degrade the overall system performance. Notably, for the semi‐hexagonal and parabolic metasurfaces, the presence of gaps appears to exert a beneficial effect in suppressing side lobe elevation, a phenomenon we ascribe to the interruption of periodic surface wave propagation along the aperture.

### 3.2 Comparison With Other Designs

A systematic comparison between the proposed metasurface and existing representative works is summarized in Table 2. Our design achieves a measured beam scanning range of ±80°, significantly wider than the ± 50° to ± 60° ranges reported in many existing conformal metasurface design. This benefits from the pattern‐reconfigurable feed antenna that maintains high aperture efficiency at large angles. The gain fluctuation remains below 3 dB across the entire scanning range, which is comparable to the best reported results and superior to designs with higher gain variation, while employing a much simpler and more cost‐effective architecture. Unlike designs relying on complex curved substrates or intricate varactor‐based tuning, our design uses standard rigid PCB technology, enabling low‐cost mass production; the bamboo‐slip‐inspired splicing also allows easy replacement of damaged subarrays, reducing maintenance costs. Moreover, by mounting identical subarrays onto different 3D‐printed skeletons, our design can realize cylindrical, semi‐hexagonal, and parabolic surfaces—a versatility not offered by designs limited to a single fixed curvature or those requiring complex flexible materials.

**TABLE 2:** Comparison of the proposed metasurface with existing representative works.

Reference	Freq. (GHz)	Scanning Range	Gain Fluctuation	Manufacturing Cost	Curvature adaptation capability
[56]	9.3–10.5	−50°–50°	∼2.9 dB	High	Fixed
[33]	4.74–5.12	−60°–60°	NaN	Medium	Fixed
[43]	3.0–3.4	−60°–60°	∼3 dB	High	Dynamic
[57]	11.8‐12.0	−50°–50°	∼4 dB	Medium	Fixed
[58]	4.35	0°	NaN	High	Fixed
This Work	5.77‐5.89	−80°–80°	< 3 dB	Low	High

It should be noted that, in addition to beam steering, our conformal reconfigurable metasurface holds promise for various dynamic wavefront manipulation functionalities, such as multi‐beam generation, beam focusing, and vortex beam generation carrying orbital angular momentum. Conformal arrays inherently provide wide‐angle beam coverage and aerodynamic benefits. Nevertheless, to achieve favorable performance in implementing such wavefront manipulations on conformal surfaces, critical issues must be carefully addressed, including the extra phase compensation induced by the curved geometry, polarization mismatch, feed‐metasurface synergy, and the gaps and coupling effects at the junctions between subarrays.

## 4 Conclusions

We propose a bamboo‐slip‐inspired conformal reconfigurable transmission metasurface system. By designing different structural skeletons, various conformal metasurfaces of diverse shapes and sizes can be constructed using sub‐arrays with the shape of bamboo‐slip. This metasurface can achieve wide‐angle beam coverage in platform scenarios of different shapes. To achieve this purpose, we respectively designed a transmitting 1‐bit phase‐reconfigurable meta‐atom and a feed antenna with pattern switching. The on‐off state of the PIN diode in this meta‐atom generates a phase difference of 180° ± 10° at the operating frequency of 5.8 GHz. Meanwhile, the meta‐atom is longitudinally composed into some 12 × 1 subarrays. The switching of the states of the two PIN diodes on the feed antenna can respectively generate the main beam radiation regions in the directions of 0° and ± 40°. The bamboo‐slip‐inspired conformal reconfigurable transmission metasurface can fix the subarray into the designed curved surface through a 3D‐printed skeleton. Based on the principle of beam scanning, the phase compensation distribution of conformal metasurfaces is calculated, and the working state of the paranoid voltage control unit is output through the FPGA control board, which can achieve the regulation of the direction of the transmitted wave. A 25 × 12 prototype was fabricated to validate the radiation characteristics of the conformal reconfigurable metasurface. The experimental results show that the cylindrical conformal metasurface can achieve wide‐angle beam scanning within the range of ‐80° to 80°. At the same time, the beam scanning performance of other shapes such as semi‐hexagonal and parabolic surfaces that can be spliced was also tested.

The bamboo‐slip‐inspired conformal reconfigurable metasurface can adapt to various application requirements, making metasurfaces have wide application potential in fields such as communication and radar systems with different environments. In addition, by using multiple subarrays to form a curved surface array, not only can the existing printed circuit board (PCB) technology be fully utilized for processing, but also a curved surface array with a larger curvature can be achieved.

## Author Contributions

**Dexiao Xia**: software, formal analysis, visualization. **Qifan Li**: conceptualization, methodology, writing – original draft. **Qiang Feng**: conceptualization, validation, writing – review and editing. **Shaofeng Zhang**: data curation, investigation, writing – original draft. **Guangyao Liu**: conceptualization, software, funding acquisition. **Xiangjin Ma**: data curation, investigation, visualization. **Yajie Mu**: validation, methodology, resources. **Jiaqi Han**: conceptualization, project administration, writing – review and editing. **Haixia Liu**: investigation, validation, writing – review and editing. **Long Li**: conceptualization, supervision, project administration, writing – review and editing.

## Conflicts of Interest

The authors declare no conflicts of interest.

## Supporting information




**Supporting File**: advs77188‐sup‐0001‐SuppMat.docx.

## Data Availability

The data that support the findings of this study are available from the corresponding author upon reasonable request.

## References

[1] C.‐M. Nieh , C. Wei , and J. Lin , “Concurrent Detection of Vibration and Distance Using Unmodulated CW Doppler Vibration Radar with an Adaptive Beam‐Steering Antenna,” IEEE Transactions on Microwave Theory and Techniques 63, no. 6 (2015): 2069–2078, 10.1109/tmtt.2015.2422692.

[2] M. He and J. Ni , “Effects of Phase Shifter Quantization on Secret Millimeter‐Wave Communication Systems with Phased‐Array Antennas,” IEEE Transactions on Vehicular Technology 73, no. 11 (2024): 17265–17275, 10.1109/tvt.2024.3429232.

[3] W. Xu , J. Hu , P. Huang , W. Tan , and Y. Dong , “Multiaperture Antenna Architecture Design for Azimuth Uniform Sampling in High‐Resolution Wide‐Swath SAR,” IEEE Antennas and Wireless Propagation Letters 19, no. 6 (2020): 1042–1046, 10.1109/lawp.2020.2988819.

[4] D. Liang , H. T. Hui , T. S. Yeo , and B. K. Li , “Stacked Phased Array Coils for Increasing the Signal‐to‐Noise Ratio in Magnetic Resonance Imaging,” IEEE Transactions on Biomedical Circuits and Systems 7, no. 1 (2013): 24–30, 10.1109/tbcas.2012.2194144. 23853276

[5] A. K. Agrawal and E. L. Holzman , “Beamformer Architectures for Active Phased‐Array Radar Antennas,” IEEE Transactions on Antennas and Propagation 47, no. 3 (1999): 432–442, 10.1109/8.768777.

[6] J. Xiao , S. Liao , Q. Xue , and W. Che , “Millimeter‐Wave 1‐D Wide‐Angle Scanning Pseudo‐Curved Surface Conformal Phased Array Antennas Based on Elongated Planar Aperture Element,” IEEE Transactions on Antennas and Propagation 71, no. 4 (2023): 3731–3735, 10.1109/tap.2023.3242112.

[7] T. J. Cui , M. Q. Qi , X. Wan , J. Zhao , and Q. Cheng , “Coding Metamaterials, Digital Metamaterials and Programmable Metamaterials,” Light: Science & Applications 3, no. 10 (2014): e218–e218, 10.1038/lsa.2014.99.

[8] L. Zhu , J. Han , Z. Zheng , et al., “Hierarchical Reconfigurable Metasurface Based on Scenario‐Guided Functional Modules and Programmable Core,” Advanced Science 13, no. 28 (2026): 24154, 10.1002/advs.202524154. PMC13185813 41823057

[9] S. Zhang , X. Ma , J. Han , et al., “Dynamic Impedance Embedding Amplitude‐/Phase‐Independent Programmable Metasurface for SWIPT Beamforming,” IEEE Transactions on Microwave Theory and Techniques 74, no. 2 (2026): 1861–1873, 10.1109/TMTT.2025.3627908.

[10] F.‐A. Li , G. Xiao , P. Li , and W. Zhu , “Reconfigurable Metasurfaces and Meta‐Devices with Multi‐Domain Multiplexing,” Device 4 (2026): 101175, 10.1016/j.device.2026.101175.

[11] Q. Li , Q. Feng , Z. Zheng , et al., “Phase‐Only Beamforming Metasurfaces with Double‐Phase Hologram Method,” IEEE Transactions on Microwave Theory and Techniques 74, no. 3 (2026): 2430–2445, 10.1109/TMTT.2026.3654185.

[12] L. Zhang , X. Q. Chen , S. Liu , et al., “Space‐Time‐Coding Digital Metasurfaces,” Nature Communications 9, no. 1 (2018): 4334, 10.1038/s41467-018-06802-0. PMC6194064 30337522

[13] Y. Mu , J. Han , L. Zhu , et al., “A Low‐Profile Self‐Stealth Programmable Metasurface with in‐Band and out‐of‐Band RCS Reduction,” Research 8 (2025): 1017, 10.34133/research.1017. PMC13248699 42273710

[14] Y. Mu , J. Han , H. Xue , et al., “Electromagnetic All‐in‐One Radiation‐Scattering Reconfigurable Intelligent Metasurface,” National Science Review 12, no. 12 (2025): nwaf470, 10.1093/nsr/nwaf470. PMC12704106 41403693

[15] eds. L. Li , Y. Shi , and T. J. Cui , in Electromagnetic Metamaterials and Metasurfaces: From Theory to Applications (Springer Nature Singapore, 2024), 10.1007/978-981-99-7914-1.

[16] X. Wang , J. Q. Han , G. X. Li , et al., “High‐Performance Cost Efficient Simultaneous Wireless Information and Power Transfers Deploying Jointly Modulated Amplifying Programmable Metasurface,” Nature Communications 14, no. 1 (2023): 6002, 10.1038/s41467-023-41763-z. PMC10522703 37752144

[17] D. X. Xia , J. Q. Han , Y. J. Mu , et al., “Adaptive Wireless‐Powered Network Based on CNN Near‐Field Positioning by a Dual‐Band Metasurface,” Nature Communications 15, no. 1 (2024): 10358, 10.1038/s41467-024-54800-2. PMC11604785 39609441

[18] S. Zhang , H. Xue , Z. Zheng , et al., “Obstacle‐Immune Microwave Wireless Power Transfer System Based on Amplitude‐Dependent Caustic Metasurface,” Advanced Science 12, no. 39 (2025): 10070, 10.1002/advs.202510070. PMC12533293 40757488

[19] M. Chang , Y. Mu , J. Han , et al., “Tailless Information–Energy Metasurface,” Advanced Materials 36, no. 21 (2024): 2313697, 10.1002/adma.202313697. 38364255

[20] Y. Saifullah , Y. He , A. Boag , G. Yang , and F. Xu , “Recent Progress in Reconfigurable and Intelligent Metasurfaces: A Comprehensive Review of Tuning Mechanisms, Hardware Designs, and Applications,” Advanced Science 9, no. 33 (2022): 2203747, 10.1002/advs.202203747. PMC9685480 36117118

[21] Z. Wei , H. Xue , Y. Li , S. Zhao , Z. Chen , and L. Li , “A Hybrid RF and Solar Integrated Energy Harvesting System Using Optically Transparent Metasurface,” IEEE Transactions on Antennas and Propagation 73, no. 2 (2025): 920–927, 10.1109/TAP.2024.3514200.

[22] H. Li , G. Wang , G. Hu , T. Cai , C. Qiu , and H. Xu , “3D‐Printed Curved Metasurface with Multifunctional Wavefronts,” Advanced Optical Materials 8, no. 15 (2020): 2000745, 10.1002/adom.202000129.

[23] L. Bao , L. L. Zhu , X. Y. Guo , et al., “Transparent and Conformal Directional Janus Metasurface with Joint Amplitude and Phase Modulations,” Laser & Photonics Reviews 19, no. 18 (2025): 2500340, 10.1002/lpor.202500340.

[24] E. Wen , X. Yang , and D. F. Sievenpiper , “Real‐Data‐Driven Real‐Time Reconfigurable Microwave Reflective Surface,” Nature Communications 14, no. 1 (2023): 3161, 10.1038/s41467-023-43473-y. PMC10676374 38007465

[25] Y. Fu , X. Huang , Y. Luo , et al., “A Multi‐Polarization Multi‐Band Reconfigurable Cloaking‐Illusion Metasurface,” Advanced Optical Materials 13, no. 4 (2025): 2402345, 10.1002/adom.202402381.

[26] S. M. Kamali , A. Arbabi , E. Arbabi , Y. Horie , and A. Faraon , “Decoupling Optical Function and Geometrical Form Using Conformal Flexible Dielectric Metasurfaces,” Nature Communications 7, no. 1 (2016): 11673, 10.1038/ncomms11618. PMC4874029 27193141

[27] D. K. Nikolov , A. Bauer , F. Cheng , H. Kato , A. N. Vamivakas , and J. P. Rolland , “Metaform Optics: Bridging Nanophotonics and Freeform Optics,” Science Advances 7, no. 18 (2021): abj9137, 10.1126/sciadv.abe5112. PMC8087415 33931445

[28] M. Gal‐Katziri , A. Fikes , and A. Hajimiri , “Flexible Active Antenna Arrays,” npj Flexible Electronics 6, no. 1 (2022): 15, 10.1038/s41528-022-00218-z.

[29] E. Gupta , C. Bonner , N. Lazarus , M. S. Mirotznik , and K. J. Nicholson , “Multiaxis Manufacture of Conformal Metasurface Antennas,” IEEE Antennas and Wireless Propagation Letters 22, no. 11 (2023): 2629–2633, 10.1109/lawp.2023.3282556.

[30] Z. Li , K. Wang , Y. Lv , S. Qian , X. Zhang , and X. Cui , “Wing Conformal Load‐Bearing Endfire Phased Array Antenna Skin Technology,” IEEE Transactions on Antennas and Propagation 71, no. 3 (2023): 2064–2069, 10.1109/tap.2022.3231081.

[31] H. Li , C. Ma , F. Shen , et al., “Wide‐Angle Beam Steering Based on an Active Conformal Metasurface Lens,” IEEE Access 7 (2019): 185264–185272, 10.1109/access.2019.2960639.

[32] X. Yang , S. Chen , Y. Chen , et al., “Combining Pancharatnam‐Berry Phase and Spherical Conformal Transmitarray for High‐Efficiency Beam Focusing,” IEEE Transactions on Antennas and Propagation 72, no. 11 (2024): 8452–8465, 10.1109/tap.2024.3449077.

[33] Z. Wang , Y. Liu , and Y. Dong , “Beam‐Switchable Digital Conformal Array with Metasurface Phase Compensation,” IEEE Transactions on Antennas and Propagation 73, no. 3 (2025): 1523–1536, 10.1109/tap.2024.3508138.

[34] Y. Xia , B. Muneer , and Q. Zhu , “Design of a Full Solid Angle Scanning Cylindrical‐and‐Conical Phased Array Antennas,” IEEE Transactions on Antennas and Propagation 65, no. 9 (2017): 4645–4655, 10.1109/tap.2017.2730241.

[35] C. Fan , W. Zhao , K. Chen , J. Zhao , T. Jiang , and Y. Feng , “Optically Transparent Conformal Reflectarray with Multi‐Angle Microwave Efficient Scattering Enhancement,” Advanced Optical Materials 12, no. 18 (2024): 2400151, 10.1002/adom.202400151.

[36] G. Sun , J. Wang , S. Xing , D. Huang , D. Feng , and X. Wang , “A Flexible Conformal Multifunctional Time‐Modulated Metasurface for Radar Characteristics Manipulation,” IEEE Transactions on Microwave Theory and Techniques 72, no. 7 (2024): 4294–4308, 10.1109/tmtt.2023.3337646.

[37] D. Gao , R. Yang , C. Gu , et al., “Conformal Cassegrain Reflecting Systems Using Meta‐Surfaces,” Journal of Physics D: Applied Physics 52, no. 23 (2019): 235301, 10.1088/1361-6463/ab0dd7.

[38] P.‐Y. Qin , L. Song , and Y. J. Guo , “Beam Steering Conformal Transmitarray Employing Ultra‐Thin Triple‐Layer Slot Elements,” IEEE Transactions on Antennas and Propagation 67, no. 8 (2019): 5390–5398, 10.1109/tap.2019.2918496.

[39] Y. Wang , Q. Feng , X. Kong , H. Liu , J. Han , and L. Li , “Multifeed Beam‐Switchable Cylindrical Conformal Holographic Metasurface Antenna,” IEEE Antennas and Wireless Propagation Letters 23, no. 3 (2024): 970–974, 10.1109/lawp.2023.3340682.

[40] X. Wang , P.‐Y. Qin , L.‐Z. Song , R. Jin , and Y. J. Guo , “Tightly Coupled Huygens Element‐Based Conformal Transmitarray for E‐Band Airborne Communication Systems,” IEEE Transactions on Antennas and Propagation 71, no. 3 (2023): 2467–2475, 10.1109/tap.2023.3241044.

[41] Z.‐H. Fu , X.‐S. Yang , and B.‐Z. Wang , “Low‐Cost Conformal Transmit‐Reflect‐Array Antenna for Unmanned Aerial Vehicle Relay Communications,” IEEE Transactions on Antennas and Propagation 73, no. 5 (2025): 2950–2960, 10.1109/tap.2024.3518632.

[42] L.‐Z. Song , P.‐Y. Qin , and Y. J. Guo , “A High‐Efficiency Conformal Transmitarray Antenna Employing Dual‐Layer Ultrathin Huygens Element,” IEEE Transactions on Antennas and Propagation 69, no. 2 (2021): 848–858, 10.1109/tap.2020.3016157.

[43] F. Li , T. Pan , W. Li , et al., “Flexible Intelligent Microwave Metasurface with Shape‐Guided Adaptive Programming,” Nature Communications 16, no. 1 (2025): 3161, 10.1038/s41467-025-58249-9. PMC11965423 40175349

[44] S. Aksu , M. Huang , A. Artar , et al., “Flexible Plasmonics on Unconventional and Nonplanar Substrates,” Advanced Materials 23, no. 38 (2011): 4422–4430, 10.1002/adma.201102430. 21960478

[45] Z. Guanxing , Z. Liu , W. Deng , and W. Zhu , “Reconfigurable Metasurfaces with Mechanical Actuations: towards Flexible and Tunable Photonic Devices,” Journal of Optics 23, no. 1 (2021): 013001, 10.1088/2040-8986/abcc52.

[46] K. J. Nicholson , T. C. Baum , and K. Ghorbani , “Conformal Voronoi Metasurface Antenna Embedded in a Composite Structural Laminate,” IEEE Transactions on Antennas and Propagation 69, no. 7 (2021): 3717–3725, 10.1109/tap.2020.3044663.

[47] W. Tao , J. Haas , Y. Wu , et al., “Dynamic Tuning of Surface Lattice Resonances via Mechano‐Optical Coupling in Flexible Nanoring Metasurfaces,” Applied Physics Letters 126, no. 20 (2025): 034102, 10.1063/5.0267314.

[48] J. Liu , S. Jiang , W. Xiong , C. Zhu , K. Li , and Y. Huang , “Self‐Healing Kirigami Assembly Strategy for Conformal Electronics,” Advanced Functional Materials 32, no. 12 (2022): 2109214, 10.1002/adfm.202109214.

[49] E. Bermúdez‐Ureña and U. Steiner , “Self‐Rolled Multilayer Metasurfaces,” ACS Photonics 6, no. 9 (2019): 2198–2204, 10.1021/acsphotonics.9b00816.

[50] S. Jiang , J. Liu , W. Xiong , et al., “A Snakeskin‐Inspired, Soft‐Hinge Kirigami Metamaterial for Self‐Adaptive Conformal Electronic Armor,” Advanced Materials 34, no. 31 (2022): 2204091, 10.1002/adma.202204091. 35680159

[51] Y. Zhou , S. Wang , J. Yin , et al., “Flexible Metasurfaces for Multifunctional Interfaces,” ACS Nano 18, no. 4 (2024): 2685–2707, 10.1021/acsnano.3c09310. 38241491

[52] Y. L. Fan , X. Q. Lin , and S. L. Liu , “A Broadband Bifocal Conformal Transmitarray Antenna with Wide Scanning Angles,” Microwave and Optical Technology Letters 64, no. 10 (2022): 1800–1808, 10.1002/mop.33366.

[53] X. Liu , Z. Yan , E. Wang , X. Zhao , T. Zhang , and F. Fan , “Multibeam Forming with Arbitrary Radiation Power Ratios Based on a Conformal Amplitude–Phase‐Controlled Metasurface,” IEEE Transactions on Antennas and Propagation 71, no. 4 (2023): 3707–3712, 10.1109/TAP.2023.3242836.

[54] A. Clemente , L. Dussopt , R. Sauleau , P. Potier , and P. Pouliguen , “1‐Bit Reconfigurable Unit Cell Based on PIN Diodes for Transmit‐Array Applications in $X$‐Band,” IEEE Transactions on Antennas and Propagation 60, no. 5 (2012): 2260–2269, 10.1109/tap.2012.2189716.

[55] J. Tian , S. Li , C. He , and W. Zhu , “Wideband Transmissive Programmable Metasurface for Adaptive Millimeter‐Wave Beamforming,” Laser & Photonics Reviews 19, no. 3 (2025): 2401333, 10.1002/lpor.202401333.

[56] H. Chen , T. Liu , M. Chen , et al., “Wide‐Angle Conformal Active Metasurface for Dynamic Beam Steering and Orbital Angular Momentum Generation,” Laser & Photonics Reviews 20, no. 3 (2026): 01500, 10.1002/lpor.202501500.

[57] J. Liao , C. Ji , L. Yuan , et al., “Polarization‐Insensitive Metasurface Cloak for Dynamic Illusions with an Electromagnetic Transparent Window,” ACS Applied Materials & Interfaces 15, no. 13 (2023): 16953–16962, 10.1021/acsami.2c21565. 36867759

[58] G. Bai , J. Liao , C. Huang , et al., “Rapid Excitation Retrieval for Large‐Scaled Antenna Arrays Using Nonconvex Optimization and Phaseless Measurements,” IEEE Transactions on Antennas and Propagation 74, no. 1 (2026): 1233–1238, 10.1109/TAP.2025.3617227.

