# Supplementary material for: Bamboo‐Slip‐Inspired Conformal Reconfigurable Metasurfaces
Source: Adv Sci (Weinh). 2026 Aug 13;13(58):e77188. doi: 10.1002/advs.77188 (PMC13472105; doi:10.1002/advs.77188)
Supplement: Supplementary file 1 [file ADVS-13-e77188-s001.docx]

Supporting Information

Bamboo-Slip-Inspired Conformal Reconfigurable Metasurfaces

Qifan Li ^1+^, Shaofeng Zhang ^1+^, Qiang Feng ^1+^, Dexiao Xia ^1^, Yajie Mu ^1^, Xiangjin Ma ^1^, Jiaqi Han ^1^*, Haixia Liu ^1^, Guangyao Liu ^2^*, and Long Li ^1^*

^1^Key Laboratory of High Speed Circuit Design and EMC of Ministry of Education, School of Electronic Engineering, Xidian University, Xi'an 710071, China

^2^State Key Laboratory of Terahertz and Millimeter Waves and Department of Electrical Engineering, City University of Hong Kong, Hong Kong SAR, China.

*Corresponding authors. E-mail: [jqhan@xidian.edu.cn](mailto:jqhan@xidian.edu.cn), guangyliu7@cityu.edu.hk and lilong@mail.xidian.edu.cn

^+^Q.L., S.Z., and Q. F. contribute equally to this work and should be considered co-first authors.

This supporting information includes:

**Supplementary Notes 1-14**

**Figures S1-S16**

**Tables S1-S4**

**Supplementary Note 1. Switching of the feed antenna radiation area**

When the bamboo-slip metasurfaces are spliced to form curved surfaces, the main radiation area of the feed antenna needs to change according to the variation of the scanning angle. As shown in **Figure S1**, the main radiation direction of the feed antenna depends on the states of the two PIN diodes. The main radiation direction of the feed will affect the amplitude and phase of the electromagnetic waves received by the meta-atoms on the conformal metasurface. For instance, if we want the conformal metasurface to generate a beam within *θ* = ±45°, the feed source should be in an Off-Off state. At this point, the meta-atoms in the middle region of the conformal metasurface will be activated. If the meta-atoms in this region contribute the most to the beam within *θ* = ±45°, then the radiation efficiency of the beam is the highest. When we want the conformal metasurface to generate a beam of *θ* = 45° to 80°, to ensure the radiation efficiency of the beam, the state of the feed antenna should be switched to On-Off. At this point, the right half of the meta-atoms on the conformal metasurface will be activated. Meanwhile, the phase distribution across the conformal metasurface remains largely unchanged. Finally, when we want the conformal metasurface to generate a beam of *θ* = -45° to -80°, based on the above analysis, the state of the feed antenna should be switched to Off-On.

**Figure S1.** Switching of the feed antenna radiation area. (a) Amplitude. (b) Phase.

The metasurfaces and the feed antenna operate independently but work in a coordinated manner. The FPGA control board supplies power to both the metasurface elements and the feed antenna, individually controlling the states of their respective PIN diodes. The metasurface elements calculate and load the corresponding phase coding according to the desired beam direction, while the feed antenna switches to the appropriate radiation state based on the beam steering angle.

**Supplementary Note 2. Design Principle of the Meta-atom**

Meta-atom adopted are classic U-shaped and O-shaped slot structures. The radiation structure layer (upper patch) and the receiving structure layer (lower patch) are shown in **Figures S2a** and **S2b**, respectively. The side view of meta-atom is shown in the **Figures S2c**. Its design geometric parameters are shown in **Table S1**. The two metal patches are connected by a metal via. At both ends of the fine metal wire in the middle of the O-shaped slot, one SMP1340 diode is embedded at each end. The PIN diode is modeled as a series equivalent circuit, as shown in **Figures S2d**. In the On state, it consists of a resistance R = 0.85 Ω and an inductance L = 30 pH. In the Off state, it is represented by a capacitance C = 40 fF and an inductance L = 30 pH. Using a dual-polarity power supply, when the upper patch is biased at +5 V, PIN-1 conducts while PIN-2 is cut off. Conversely, when the upper patch is biased at -5 V, PIN-2 conducts while PIN-1 is cut off, as shown in **Figures S2e**.

**Figure S2.** (a) the radiation structure layer (upper patch), (b) the receiving structure layer (lower patch) and (c) the side view of the meta-atom. (d) Equivalent circuit of PIN diode. (e) Equivalent circuit of the meta-atom.

TABLE S1

Geometric Parameters of Meta-Atom

| Parameters | *p* | *h_1_* | *h_2_* | *a* | *b_1_* |
| --- | --- | --- | --- | --- | --- |
| Value | 25 mm | 1.524 mm | 0.4 mm | 11.7 mm | 4.2 mm |
| Parameters | *b_2_* | *b_3_* | *b_4_* | *b_5_* | *w_1_* |
| Value | 6.6 mm | 5.0 mm | 6.3 mm | 8.75 mm | 0.8 mm |
| Parameters | *w_2_* | *l_1_* | *l_2_* | *r_1_* | *α* |
| Value | 0.2 mm | 2.2 mm | 6.2 mm | 6.0 mm | 30 deg |

Full-wave simulation was carried out using the commercial simulation software ansys HFSS, and the meta-atom properties under periodic boundary conditions under normal incidence and oblique incidence were simulated respectively. Under the case of oblique incidence, the amplitude phase responses of the unit when it was oblique incident at 20° and 40° were simulated respectively, as shown in **Figures S3a-d**. The maximum insertion loss of this unit within 40° was only 1.21 dB, and the phase difference between the two states was approximately 180°, with an error not exceeding 2°.

**Figure S3.** (a) amplitude and (b) phase characteristics when oblique incident wave at 20°. (c) amplitude and (d) phase characteristics when incident wave at 40°.

**Supplementary** **Note 3. Design and Operating Principle of the Feed Antenna**

The pattern reconfigurable antenna consists of a vertical dipole antenna and two active guiding elements at the top, as shown in **Figure S4**. The specific geometric size parameters of the pattern reconfigurable antenna are shown in **Table S2**. The 3D radiation pattern of the pattern reconfigurable antenna in different states of the PIN diode are shown in **Figure S5**. When the state is Off-Off, the radiation direction is along the principal axis (*θ* = 0°), while in the other two states, the radiation direction deviates from the principal axis by 40° (*θ* = ±40°). By switching the above three states, the purpose of switching the radiation region can be achieved.

**Figure S4.** 3D and 2D structures of the feed antenna design.

TABLE S2

Geometric Parameters of Feed Antenna

| Parameter | *l_a_*_1_ | *l_a_*_2_ | *l_a_*_3_ | *l_a_*_4_ | *l_a_*_5_ |
| --- | --- | --- | --- | --- | --- |
| Value | 5.5 mm | 6.8 mm | 3.8 mm | 4.0 mm | 16.0 mm |
| Parameter | *w_a_*_1_ | *w_a_*_2_ | *w_a_*_3_ | *s_a_*_1_ | *s_a_*_2_ |
| Value | 1.4 mm | 4.0 mm | 1.2 mm | 11.4 mm | 2.2 mm |
| Parameter | *s_a_*_3_ | *h_a_*_1_ | *h_a_*_2_ | *p_a_* |  |
| Value | 1.0 mm | 12.0 mm | 1.0 mm | 40.0 mm |  |

The proposed pattern reconfigurable antenna can be conceptually regarded as a special type of Yagi-Uda antenna as shown in **Figure S5**. In this configuration, the vertical dipole antenna functions as the driven element, while the two active guiding elements at the top serve as either directors or reflectors, depending on the PIN diode states. Specifically, when the PIN diode is ON state (conducting), the guiding element behaves as a reflector; when the PIN diode is OFF state (non-conducting), the guiding element behaves as a director. Therefore, the radiation pattern reconfiguration is achieved by dynamically altering the parasitic loading of the two guiding elements. By switching the PIN diodes between ON and OFF states, each guiding element alternates between reflecting and directing functions, thereby changing the overall array configuration and the resulting radiation pattern.

As for the factors influencing the deflection angle, the primary parameters include the spacing between the two active guiding elements, the length of each active guiding element, and the width of each active guiding element. These geometrical parameters determine the phase difference and coupling between the driven element and the parasitic elements, which collectively govern the direction of the main beam. In our design, the Off-Off state yields radiation along the principal axis (θ = 0°), while the other two available states (On-Off and Off-On) produce beam deflections of θ = ±40°, respectively.

**Figure S5.** 3D Radiation pattern in different states.

**Supplementary** **Note 4. Phase compensation based on generalized Snell’s law**

Two mainstream methods, direct phase shift and phase gradient, are commonly used for the analysis of phase distribution in planar metasurfaces. The former is based on geometric optical ray tracing, while the latter is based on the generalized Snell’s law, which was proposed by Professor Capasso in 2011 ^[1]^. For surfaces that have analytical solutions, using the phase gradient method to obtain the phase compensation of metasurfaces has the advantages of simplicity and intuitiveness.

**a	b**

**Figure S6.** Generalized Snell's law on (a) general curved surface and (b) cylindrical surface.

The surface Generalized Snell’s law transmission theorem for arbitrary surfaces, as shown in **Figure S6a**, can be written as

$$\hat{n}\times(\vec{k}_{t}-\vec{k}_{i})=\hat{n}\times\nabla\Phi$$

Where, $\hat{n}$ is the unit normal vector of the surface, $\vec{k}_{i}$ and $\vec{k}_{t}$ are the wave vectors of the incident and transmitted waves, respectively, and $\nabla\Phi$ is the phase gradient on the surface. Establishing a moving frame on the surface, denoted as $\{\hat{n},\hat{t}_{1},\hat{t}_{2}\}$, where $\hat{t}_{1}$ and $\hat{t}_{2}$ represent the unit tangent vectors of two orthogonal curves on the surface. By taking the cross product of both sides of equation (S1) with the unit vector $\hat{t}_{1}$, we have

$$\hat{t}_{1}\times\left[\hat{n}\times(\vec{k}_{t}-\vec{k}_{i})\right]=\hat{t}_{1}\times(\hat{n}\times\nabla\Phi)$$

By utilizing the double cross product vector identity and taking into account $\hat{t}_{1}\cdot\hat{n}=0$, equation (S2) can be simplified to

$$\hat{t}_{1}\cdot(\vec{k}_{t}-\vec{k}_{i})=\hat{t}_{1}\cdot\nabla\Phi$$

Similarly, it can be obtained

$$\hat{t}_{2}\cdot(\vec{k}_{t}-\vec{k}_{i})=\hat{t}_{2}\cdot\nabla\Phi$$

Meanwhile, due to $\hat{t}\cdot\nabla\Phi=\partial\Phi/\partial l$, equations (S3) and (S4) can be written as

$$\left\{\begin{matrix} \hat{t}_{1}\cdot(\vec{k}_{t}-\vec{k}_{i})=\frac{\partial\Phi}{\partial l_{1}} \\ \hat{t}_{2}\cdot(\vec{k}_{t}-\vec{k}_{i})=\frac{\partial\Phi}{\partial l_{2}} \end{matrix}\right.$$

Equation (S5) is the component form of the generalized Snell’s Law for surfaces, where $\partial\Phi/\partial l$ represents the directional derivative along the curve $l$. According to **Figure S6b**, the unit tangent vectors of the two orthogonal coordinate curves on the cylindrical surface are and $\hat{t}_{2}=(0,0,1)$ respectively. Assuming the source of the emitted spherical wave is located at the point *O*, the wave vector of the incident wave is given by

To generate a plane wave with a direction angle of, the wave vector of the transmitted wave is

By substituting the $\hat{t}_{1}$ and $\hat{t}_{1}$, as well as equations (S6) and (S7), into equation (S5), we have

Equation (S8) is the phase gradient of the cylinder surface which is incidence by a spherical wave and transmitting a plane wave with a direction angle. As the required distribution is the phase shift distribution, it is necessary to integrate equation (S8). The result after integration, which is the desired phase compensation in this paper, can be written as

It is worth noting that the value of the phase gradient is unique, while the compensating phase is not unique, differing by a constant value *C*. This is consistent with practical situations, as it is the phase difference rather than the absolute phase that determines the direction of beam radiation. For convenience, the constant *C* can be set to zero.

In addition, we also calculated the phase compensation for the plane surface and the parabolic cylinder surface, shown in **Figure S7a and S7b** respectively.

According to **Figure S7a**, the unit tangent vectors of the two orthogonal coordinate curves on the plane surface are $\hat{t}_{1}=(1,0,0)$ and $\hat{t}_{2}=(0,1,0)$ respectively. Assuming the source of the emitted spherical wave is located at the point *-F*, the wave vector of the incident wave is given by

$$\vec{k}_{i}=\frac{k}{\sqrt{x^{2}+y^{2}+F^{2}}}(x,y,F)$$

**a	b**

**Figure S7.** Generalized Snell's law on (a) plane surface and (b) parabolic cylinder surface.

By substituting the $\hat{t}_{1}$ and $\hat{t}_{1}$, as well as equations (S10) and (S7), into equation (S5), we have

The result after integration, which is the desired phase compensation in this paper, can be written as

According to **Figure S7b**, the unit tangent vectors of the two orthogonal coordinate curves on the parabolic cylinder surface are $\hat{t}_{1}=\frac{1}{\sqrt{1+\frac{x^{2}}{4p^{2}}}}(1,-\frac{x}{2p},0)$ and $\hat{t}_{2}=(0,0,1)$ respectively. Assuming the source of the emitted spherical wave is located at the point *O*, the wave vector of the incident wave is given by

$$\vec{k}_{i}=\frac{k}{\Delta}(x,F-\frac{x^{2}}{4p},z)$$

where $\Delta=\sqrt{x^{2}+z^{2}+(F-\frac{x^{2}}{4p})^{2}}$. By substituting the $\hat{t}_{1}$ and $\hat{t}_{1}$, as well as equations (S13) and (S7), into equation (S5), we have

The result after integration can be written as

**Supplementary Note 5. The equations of semi-hexagonal prisms and parabolic prisms**

As shown in the Cartesian coordinate system in **Figure S8a and S8b**, the generatrix direction of the one-dimensional semi-hexagonal and parabolic are all along the *x*-direction. The equation of the conformal semi-hexagonal prisms shown in **Figure S8a** is

$\left\{\begin{matrix} z=\frac{\sqrt{3}}{2}D,if\left|y\right|\leq\frac{1}{2}D \\ z=-\sqrt{3}(y-D),ify>\frac{1}{2}D \\ z=\sqrt{3}(y+D),ify<-\frac{1}{2}D \\ -L/2<x<L/2,z>0 \end{matrix}\right.$.

Similarly, the equation of the conformal parabolic prisms shown in **Figure S8b** is

$\left\{\begin{matrix} y^{2}=-4Fz \\ x=\text{constant,} \\ z>-\frac{1}{2}F,-L/2<x<L/2, \end{matrix}\right.$.

**Figure S8.** Composition of (a) semi-hexagonal prisms and (b) parabolic prisms.

**Supplementary** **Note 6. Simulation results of radiation performance of metasurfaces**

The main lobe gain, first side lobe, back lobe and 3 dB half-power beamwidth of the conformal metasurfaces of cylindrical, semi-hexagonal prisms and parabolic are shown in **Figure S9a-c**, respectively. It can be seen that the gain fluctuations of the cylindrical metasurface and the semi-hexagonal prism metasurface are 2.53 dB and 1.75 dB respectively, with a fluctuation range of less than 3 dB. The fluctuation range of the parabolic cylinder is relatively large, with a fluctuation value of 3.65 dB. When the absolute value of the scanning angle is greater than to 60 degrees, its gain drops rapidly. The sidelobes and back lobes of all three metasurface types are approximately 10 dB lower than the main lobe. The 3 dB beamwidth broadens with the scanning angle for all three metasurface types.

**Figure S9.** Simulation results of main lobe gain, first side lobe, back lobe and 3 dB half-power beamwidth of (a) cylindrical, (b) semi-hexagonal prisms and (c) parabolic.

**Supplementary** **Note 7. Conformal metasurface prototype**

To adapt to more application scenarios and the trend of miniaturization and integration of metasurfaces, we designed different conformal shapes of metasurfaces. Through 3D printing, a structural skeleton capable of installing bamboo-slip sub-arrays was produced, as shown in **Figure S10**. **S10a:** Cylindrical structure: A 12 × 25 metasurface is formed by mounting an array of 25 bamboo-slips. The feed horn is positioned at the circle's center, 200 mm from the metasurface. **S10b:** Semi-hexagonal prismatic frame: This structure is supported by three sides of a regular hexagon. Mounting an array of 24 bamboo-slips creates a 12 × 24 metasurface. **S10c:** Parabolic structure: An array of 25 bamboo-slips forms a 12 × 25 metasurface. The feed horn is located at the parabola's focal point.

The bamboo-inspired metasurface stick sub-array is composed of 12 meta-atoms arranged longitudinally. DC line is arranged at the edge positions on both sides. The upper and lower 6 meta-atoms are routed from the left and right sides respectively, saving space. Two circular holes at the top and bottom can be fixed on the structural skeleton through m4 model screws to form conformal reconfigurable metasurfaces with different shapes.

**Figure S10.** Conformal metasurface prototype. (a) Cylindrical, (b) Hexagonal, (c) Parabolic.

**Supplementary** **Note 8. DC control board**

The DC control board provides control signals for the entire system to ensure that the conformal metasurface can precisely regulate the beam orientation of the transmitted wave. Therefore, a stable and reliable control board is the key for the entire system to operate normally. As shown in **Figure S11**, it is the physical diagram of the control board. The LED light can display the level status of all I/O ports. When the light is on, it indicates that the I/O port is at a high level; when the light is off, it indicates that the I/O port is at a low level. The function of the switch chip is to enhance the driving capability of the I/O port. The Joint Test Action Group (JTAG) port facilitates the uploading and debugging of programs. The Voltage Regulator Module (VRM) supplies a stable operating voltage to the Field Programmable Gate Array (FPGA).

**Figure S11.** A prototype of the DC control board.

**Supplementary Note 9. Experimental results of the meta-atom**

The S-parameter measurements for the fabricated reconfigurable meta-atoms were carried out using the quasi-optical free-space measurement method ^[2]^. The measurement setup is illustrated in **Figure S12**. Two spot-focusing horn lens antennas were connected to port 1 and port 2 of a vector network analyzer (VNA), respectively, with their optical axes aligned and focal points centered on the same line. The metasurface under test (MUT) was mounted on a sample holder between the two antennas, oriented perpendicular to the beam propagation direction and positioned near the focal plane. By switching the states of the entire metasurface via the FPGA, the S-parameters of the MUT under different states were measured.

The measurement scenario is shown in **Figure S12b**. The spot-focusing horn lens antennas operate in the C-band (4–8 GHz) with a center frequency of 6 GHz. At 5.8 GHz, the focal length is 210 mm, and the corresponding focal spot diameter is less than 120 mm (< 4.8 *λ*). The measured magnitude and phase of the S-parameters are presented in **Figures S12c** and **S12d**, respectively. It can be observed that within the frequency range of 5.77 GHz to 5.89 GHz, the transmission coefficient |S_21_| is greater than –2.5 dB at most frequency points. Near 5.86 GHz, |S_21_| drops below –3 dB, which is primarily attributed to the inherent low transmission coefficient of the lens antennas themselves at this frequency. Within the same frequency band, the reflection coefficient |S_11_| remains better than –2.5 dB, and the phase difference between the two states of the 1-bit meta-atom is maintained at approximately 180°, with an error within ± 2°.

Compared with the simulation results in Figure 2 of the main manuscript, the phase difference between the two states is almost identical to the simulation. The absolute phase values differ between measurement and simulation due to the additional propagation phase introduced by the lens antennas in the measurement setup; however, this does not affect the phase difference between the two states, which is the key parameter for beamforming. The measured transmission coefficients |S_21_| are slightly lower than the simulation results, which is mainly caused by the losses and attenuation introduced by the lens antennas and free-space propagation. Overall, the measurement results are in good agreement with the simulations, confirming the validity of our meta-atom design and the reliability of the fabricated prototype.

**Figure S12.** (a) Schematic, (b) photograph of the measurement setup, and (c, d) measured S-parameters of the meta-atom.

**Supplementary Note 10. Experimental results of the pattern reconfigurable antenna**

The fabricated, pattern reconfigurable antenna was tested in an anechoic chamber. The S-parameters and two-dimensional (2D) radiation pattern of the reconfigurable metasurface antenna are shown in **Figure S13**. The antenna operates at 5.7 GHz to 5.9 GHz. The reflection coefficients of the three available states within the working range are all less than -10 dB, shown in **Figure S13a**. The H-plane radiation pattern of the pattern reconfigurable antenna in different states of the PIN diode are shown in **Figure S13b**. When the state is Off-Off, the radiation direction is along the principal axis (*θ* = 0°), while in the other two states, the radiation direction deviates from the principal axis by 40° (*θ* = ±40°). By switching the above three states, the purpose of switching the radiation area can be achieved. Compared with the simulation results in the main manuscript (**Figures 3b** and **3c**), the experimental results have a certain degree of fluctuations, but the basic trend is the same as the simulation results.

**a	b**

**Figure S13.** Experimental results of the pattern reconfigurable antenna, (a) reflection coefficient and (b) 1D radiation pattern of the H plane.

**Supplementary** **Note 11. Experimental results of radiation performance of metasurfaces**

The main lobe gain, first side lobe, back lobe and 3 dB half-power beamwidth of the conformal metasurfaces of cylindrical, semi-hexagonal prisms and parabolic are shown in **Figure S14a-c**, respectively. It can be seen that the gain fluctuations of the cylindrical metasurface and the semi-hexagonal prism metasurface are 2.83 dB and 1.43 dB respectively, with a fluctuation range of less than 3 dB. The fluctuation range of the parabolic cylinder is relatively large, with a fluctuation value of 3.34 dB. When the absolute value of the scanning angle is greater than to 60 degrees, its gain drops rapidly. The sidelobes and back lobes of all three metasurface types are approximately 10 dB lower than the main lobe. The 3 dB beamwidth broadens with the scanning angle for all three metasurface types. By comparing **Figure S14** and **Figure S9**, it can be seen that the experimental results are basically consistent with the simulation results in terms of gain, beam width, side lobes and rear lobes.

**Figure S14.** Experimental results of main lobe gain, first side lobe, back lobe and 3 dB half-power beamwidth of (a) cylindrical, (b) semi-hexagonal prisms and (c) parabolic.

**Supplementary Note 12. Analysis of scanning angle ranges for different curved surfaces**

Simulation and measurement results show that the scanning angle ranges of the cylindrical and hexagonal profiles are larger than that of the parabolic profile. The fundamental cause of this phenomenon lies in the variation of the effective projected aperture as a function of the beam scanning angle θ. For any conformal array, the total physical area (A_tot_) is fixed. However, the effective area (A_eff_) that contributes to the main beam in a given direction is its projection onto the plane perpendicular to that direction. The aperture utilization efficiency is thus defined as

$$\eta_{A}\left(\theta\right)=\frac{A_{\text{eff}}(\theta)}{A_{\text{tot}}}=\frac{1\cdot L_{\text{eff}}(\theta)}{A_{\text{tot}}}=\frac{L_{\text{eff}}(\theta)}{A_{\text{tot}}}$$

For a cylindrical surface of unit height, and following the geometric relations illustrated in **Figure S15**, the effective length (L_eef_) along the curved surface can be expressed generally. To analyze the performance of the three specific conformal profiles (cylindrical, hexagonal, and parabolic), we can derive their respective L_eef_ as a function of the scanning angle *θ*.

$$L_{\text{eff,cylinder}}=R(1+\cos\theta)$$

$$L_{\text{eff,}\text{hexagonal}}=\left\{\begin{matrix} 2D_{0}\cos\theta,0\leq\left|\theta\right|<\frac{\pi}{6} \\ \sqrt{3}D_{0}\cos(\left|\theta\right|-\pi/6),\frac{\pi}{6}\leq\left|\theta\right|\leq\frac{\pi}{2} \end{matrix}\right.$$

$$L_{\text{eff,parabola}}=\left\{\begin{matrix} x_{-d}\cos\theta-y_{d}\sin\theta,-\frac{\pi}{2}\leq\theta\leq\theta_{D} \\ 2D\cos\theta,-\theta_{D}\leq\left|\theta\right|<\theta_{D} \\ x_{d}\cos\theta-y_{d}\sin\theta,\theta_{D}\leq\theta\leq\frac{\pi}{2} \end{matrix}\right.$$

where

$$\begin{matrix} x_{d}=D+2p\cot\theta,\begin{matrix} \end{matrix}x_{-d}=-D+2p\cot\theta\\ y_{d}=p\cot^{2}\theta-\frac{D^{2}}{4p},\begin{matrix} \end{matrix}\theta_{D}=\mathrm{arccot}\left(\frac{D}{2p}\right) \end{matrix}$$

The results are shown in **Figure S16**. For the cylindrical and hexagonal surfaces, the aperture utilization efficiency $\eta_{A}$ decreases slowly and monotonically as *θ* increases. This is because their curvature allows for a relatively uniform projection of the aperture onto the scanning direction.

In contrast, for the parabolic profile in our manuscript, the efficiency exhibits a much steeper decline. When the beam scans toward the edge of our parabolic surface, the effective projected aperture shrinks significantly faster than in other profiles, due to the more pronounced variation in the local surface normal along the aperture. This rapid attenuation behavior is quite similar to that of a planar array with the same physical aperture, where the gain typically drops by 3 dB or more at scanning angles around ±60°, as discussed above and illustrated in **Figure S16**. This analysis explains the sharp gain drop observed in **Figures S9** and **S14**.

**Figure S15.** Schematic of the effective aperture length L_eef_.

**Figure S16.** Aperture utilization efficiency for different curved surfaces.

**Supplementary Note 13. Beam switching time of the conformal metasurface system**

The beam switching time of the proposed conformal metasurface system is fundamentally determined by the response delays of three cascaded stages: the FPGA control logic, the analog switch array, and the PIN diode switching elements. The specific hardware parameters are listed in **Table S3**.

TABLE S3

Key Parameters of The Beam Switching Control Chain

| Component | Model | Parameter | Value |
| --- | --- | --- | --- |
| FPGA | ALINX AC7K325 (Xilinx Kintex-7 XC7K325T) | System clock frequency | 200 MHz |
|  |  | Clock period | 5 ns |
| Analog Switch | DG333ALDW-E3 | Turn-on time | 175 ns |
|  |  | Turn-off time | 145 ns |
| PIN Diode | SMP1340 | Carrier lifetime | 100 ns |

The overall beam switching time is dominated by the analog switch turn-on time (175 ns) and the PIN diode response (100 ns), with the FPGA control logic contributing negligible delay (on the order of a few clock cycles, i.e., tens of nanoseconds). Conservatively estimating the total system delay as the sum of these dominant factors, the beam switching time is approximately less than 300 ns.

**Supplementary Note 14. Power consumption analysis of the metasurface system**

The overall power consumption mainly arises from the PIN diodes (on both the metasurface and the feed antenna), the FPGA core board, and the analog switches. A component-by-component analysis is presented as follows.

1) Metasurface PIN Diode Array

In our design, each meta-atom contains two PIN diodes (SMP1340 series), and the entire prototype consists of 25 × 12 = 300 meta-atoms, resulting in a total of 600 PIN diodes. However, according to the 1-bit phase control scheme, only one of the two PIN diodes in each meta-atom is in the forward-biased (ON) state at any given time, while the other is reverse-biased (OFF). Therefore, the number of conducting diodes is 300. From the SMP1340 datasheet, Forward Voltage V_F_ is 0.85 V, Forward Current is I_F_ 10 mA. Total power consumption of the metasurface PIN diodes is

$$P_{\text{metasurface}}=300\times V_{F}\times I_{F}=300\times0.85\times10\times10^{-3}=2.55(\text{W)}$$

2) Feed Antenna PIN Diode

The feed antenna uses two PIN diodes (SMP1340). In the Off-Off state, both are OFF (negligible power). In the On-Off or Off-On states, only one diode is forward-biased, that is

$$P_{\text{feed}}=1\times V_{F}\times I_{F}=1\times0.85\times10\times10^{-3}=8.5(\text{mW)}$$

3) FPGA Control Board

The control board is based on the AV7K325 FPGA core board, which features a Xilinx Kintex-7 XC7K325T-2FFG900I FPGA. The key specifications of this FPGA include 326,080 logic cells, 840 DSP slices, 16,020 KB of Block RAM, and 16 GTX transceivers supporting up to 12.5 Gb/s.

In our application, the FPGA is primarily used to output 1-bit control signals for the metasurface (300 channels) and 2 channels for the feed antenna, operating in a low-frequency switching scenario with relatively low logic utilization. According to the Xilinx Power Estimator (XPE) tool for this device under similar low-utilization conditions, the typical power consumption is estimated to be approximately 1–2 W, including:

Core voltage (VCCINT, 1.0V): ~0.5–1.0 W

Auxiliary voltage (VCCAUX, 1.8V): ~0.1–0.2 W

I/O voltage (VCCO, 3.3V/1.8V): ~0.2–0.5 W

The control signals are distributed through 75 DG333AL analog switches. Each switch features a typical power consumption of less than 1 µW, resulting in a total power consumption of approximately 75 µW for the entire switch array, which is negligible compared to the FPGA core board.

Therefore, the total power consumption of the control board is estimated to be 0.9–1.8 W.

Based on the component specifications and the system architecture, we summarize the total power budget in **Table S4**. The metasurface PIN diode array dominates the power consumption, with a conservative worst-case estimate of 2.55 W. The FPGA core board contributes approximately 0.9–1.8W, while the 75DG333AL analog switches draw negligible power (75 × 10^-6^ W in total). The overall system power consumption is therefore estimated to be in the range of 3.5–4.4 W, which is reasonable for a 300-element active metasurface system.

TABLE S4

Total Estimated Power Budget

| Component | | Number | Power Consumption (W) |
| --- | --- | --- | --- |
| PIN Diode | Metasurface | 300 | ~2.55 |
|  | Feed Antenna | 1 | 8.5×10^-3^ |
| FPGA control board | Core Board | 1 | ~ 0.9–1.8 |
|  | Analog Switch | 75 | 75×10^-6^ |
| Total Power | | \ | ~3.5–4.4 |

**References**

1. N. Yu, P. Genevet, M. A. Kats, et al., “Light Propagation with Phase Discontinuities: Generalized Laws of Reflection and Refraction,” *Science* *334*, no. 6054 (2011): 333–337, https://doi.org/10.1126/science.1210713.

2. A. Sotiropoulos, A. Masouras, H. T. Anastassiu, V. Kostopoulos, and S. Koulouridis, “Planar Electrically Large Structures of Carbon Nanotube Films with High Absorption and Shielding Performance in X-Band,” *Sensors* *25*, no. 13 (2025): 3943, https://doi.org/10.3390/s25133943.
